# ESPERANTO: a GLP-field sEmi-SuPERvised toxicogenomics metadAta curatioN TOol

**DOI:** 10.1093/bioinformatics/btad405

**Published:** 2023-06-24

**Authors:** Emanuele Di Lieto, Angela Serra, Simo Iisakki Inkala, Laura Aliisa Saarimäki, Giusy del Giudice, Michele Fratello, Veera Hautanen, Maria Annala, Antonio Federico, Dario Greco

**Affiliations:** FHAIVE, Faculty of Medicine and Health Technology, Tampere University, Tampere 33520, Finland; FHAIVE, Faculty of Medicine and Health Technology, Tampere University, Tampere 33520, Finland; Tampere Institute for Advanced Study, Tampere, 33520, Finland; FHAIVE, Faculty of Medicine and Health Technology, Tampere University, Tampere 33520, Finland; FHAIVE, Faculty of Medicine and Health Technology, Tampere University, Tampere 33520, Finland; FHAIVE, Faculty of Medicine and Health Technology, Tampere University, Tampere 33520, Finland; FHAIVE, Faculty of Medicine and Health Technology, Tampere University, Tampere 33520, Finland; FHAIVE, Faculty of Medicine and Health Technology, Tampere University, Tampere 33520, Finland; FHAIVE, Faculty of Medicine and Health Technology, Tampere University, Tampere 33520, Finland; FHAIVE, Faculty of Medicine and Health Technology, Tampere University, Tampere 33520, Finland; Tampere Institute for Advanced Study, Tampere, 33520, Finland; FHAIVE, Faculty of Medicine and Health Technology, Tampere University, Tampere 33520, Finland; Institute of Biotechnology, Helsinki Institute of Life Sciences (HiLife), University of Helsinki, Helsinki 00790, Finland; Division of Pharmaceutical Biosciences, Faculty of Pharmacy, University of Helsinki, Helsinki 00790, Finland

## Abstract

**Summary:**

Biological data repositories are an invaluable source of publicly available research evidence. Unfortunately, the lack of convergence of the scientific community on a common metadata annotation strategy has resulted in large amounts of data with low FAIRness (Findable, Accessible, Interoperable and Reusable). The possibility of generating high-quality insights from their integration relies on data curation, which is typically an error-prone process while also being expensive in terms of time and human labour. Here, we present ESPERANTO, an innovative framework that enables a standardized semi-supervised harmonization and integration of toxicogenomics metadata and increases their FAIRness in a Good Laboratory Practice-compliant fashion. The harmonization across metadata is guaranteed with the definition of an ad hoc vocabulary. The tool interface is designed to support the user in metadata harmonization in a user-friendly manner, regardless of the background and the type of expertise.

**Availability and implementation:**

ESPERANTO and its user manual are freely available for academic purposes at https://github.com/fhaive/esperanto. The input and the results showcased in Supplementary File S1 are available at the same link.

## 1 Introduction

Public repositories store increasing volumes of toxicogenomics (TGx) data, representing a powerful and heterogenous source of prior knowledge, valuable for chemical risk assessment. In the past, however, data were generated without thinking about its potential long-term usage, resulting in poor standardization and low-quality metadata. FAIR principles guide researchers to generate reusable data, aiding science efforts to shift from traditional animal testing to alternative approaches (Saarimäki *et al.* 2021). Despite the well-defined standards for omics data representation, a large proportion of such data remains non-compliant with the FAIR principles (Wilkinson *et al.* 2016), limiting their integration and exploitation (Saarimäki *et al.* 2022). Rigorous data curation is needed to meet this challenge, harmonizing poorly standardized data to allow their integration and turn them into valuable high-quality insights (Odell et al. 2017). Several solutions have been proposed to automate the process through text mining and AI techniques (Müller *et al.* 2018, Arnaboldi *et al.* 2020, Courtot *et al.* 2022), but despite the claimed accuracy, a final validation by an experienced curator is still needed. Here, we propose ESPERANTO, a R/Shiny (Chang *et al.* 2022) application performing a streamlined semi-supervised curation of TGx metadata in compliance with Good Laboratory Practice (GLP) standards. The user is actively involved in data harmonization in a consistent framework and in the enhancement of data FAIRness, merging the advantages of both automated and manual curation approaches. Regardless of the expertise, the interactive graphical interface guides the user intuitively through the whole data curation and integration pipeline and requires almost no informatics knowledge. ESPERANTO fosters GLP principles (OECD 2021), by supplying formal guidance to reconstruct the whole pipeline behind the curation of reliable, reproducible, and high-quality data.

Although designed to run on TGx metadata, the tool can curate any health data science datasets, provided a different reference vocabulary.

## 2 Comparison with other curation tools

To the best of our knowledge, ESPERANTO is the first free available tool to allow dataset standardization and harmonization. Its closest commercial competitor, Genestack ODM (Genestack ODM | Genestack, n.d.), accepts customized vocabularies and it is pre-loaded with several validated ontologies for dataset curation, but it is not designed to adhere to GLP principles.

On the other hand, other solutions have been developed to automate data harmonization through AI techniques, but they are designed to solve slightly different tasks. In fact, most of tools focus on parsing biomedical literature by applying natural language processing techniques to automatically identify entities (i.e. signalling pathways, proteins) of interest. The results are submitted to the user for validation only through the end of the curation. Instead, with a standardized pipeline and reference vocabulary, ESPERANTO offers the user flexible and punctual curation throughout the whole pipeline. Furthermore, our method is applicable for harmonization and standardization of datasets, even in case no literature is available. A detailed comparison among the different curation tools is shown in Table 1.

**Table 1.** Comparison between ESPERANTO and other existing data curation tools.

Tool	Free license	Customizable vocabulary	Graphical user interface	Unrestricted to literature	GLP compliance	Reference
ESPERANTO	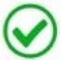	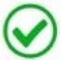	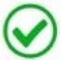	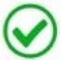	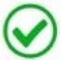	
Genestack ODM	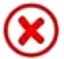	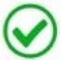	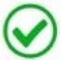	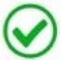	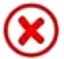	Genestack ODM | Genestack (2023 )
Textpresso Central	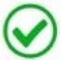	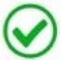	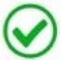	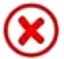	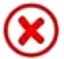	Müller *et al.* (2018)
WormBase AFP	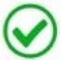	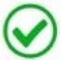	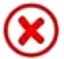	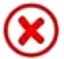	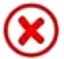	Arnaboldi *et al.* (2020)
BioSamples	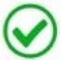	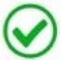	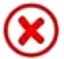	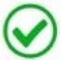	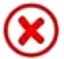	Courtot *et al.* (2022)

## 3 Methods and features

ESPERANTO ensures a streamlined and standardized TGx metadata harmonization and the construction of a customized vocabulary. Both metadata and vocabulary are essential inputs, or optionally, a previously saved curation session can be restored. An empty vocabulary is provided, but a pre-existing ontology can also be utilized as long as it is structured as described in Section 3.2. During the data curation, the software assists the decision-making of the user on each modification before implementing it on the data and recording the operation automatically. In GLP mode, the researcher is required to integrate the taken action with a justification, resulting in a meticulous GLP-compliant report. The purpose of the user guide available online at https://github.com/fhaive/esperanto is not only to instruct and support the user but also to advise on action protocols in relation to specific scenarios.

### 3.1 Curation and integration

Through word(s) matching, ESPERANTO harmonizes uncurated TGx metadata through the cross-comparison with a precompiled reference vocabulary. The tool, while originally intended for TGx metadata, has the capability to curate health data science datasets of any type as long as a distinct reference vocabulary is provided. Essentially, curation takes place in three stages: (i) consistent renaming of table columns; (ii) deletion of column duplicates; and (iii) potential editing of any remaining content. Any cross-comparison result is shown to be validated, or as an alternative, the user can edit all unique instances, storing the processed terms in a temporary vocabulary. Once multiple datasets have been curated, the tool assesses their integration, highlighting potential inconsistencies among them and/or the vocabulary through a traffic light colour system. The colour code supports the user to evaluate and mark discrepancies as ‘Issue’ to refine in a successive curation round.

**Figure 1.**
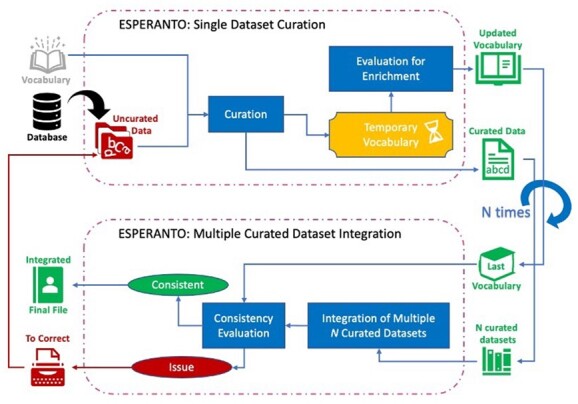
Pipeline of ESPERANTO in single and multiple dataset modes. Once the datasets of interest have been curated, they are used as input for the multiple mode. Red boxes indicate datasets or entries that need curation; dark blue represents the different processes inside ESPERANTO leading to a consistent curated dataset/entry (green). Once integration is completed and evaluated, the final output can be used for further analysis.

The effectiveness of the pipeline in metadata harmonization and integration (Fig. 1) is showcased on TGx Idiopathic Pulmonary Fibrosis datasets publicly available on Gene Expression Omnibus repository (Supplementary File S1). ESPERANTO is a powerful resource to improve FAIRness of metadata upstream of omics data preprocessing and analysis pipelines that can be easily interoperable with the tools in the Nextcast suite (Serra *et al.* 2022) (more details in Supplementary File S2). The entire design of the application was aimed to make any prerequisite knowledge unnecessary to the user for running the tool.

### 3.2 Vocabulary

The vocabulary is characterized by two linked pairs of key: synonym(s) dictionary type objects (Lai 2022). Each feature in metadata ties to all potential instances of that feature; both feature and instances are associated to their synonyms (i.e. ‘ethnicity’, ‘race’, ‘Black, White, Inuit’, ‘“B, African-American”, “Caucasian, C”’). Each round of curation offers the possibility to customize the vocabulary with new features and instances. Their evaluation follows the colour-coded mechanism established in the integration of multiple datasets.

### 3.3 GLP compliance

Being the curation influenced by the curator, it cannot be considered fully GLP compliant. With its standardized pipeline and its reference vocabulary, ESPERANTO minimizes the impact of subjectivity, at the same time, leaving the user active control over the task. Each action performed during the curation is automatically recorded (in the GLP mode together with its mandatory justification), resulting in a detailed report describing the entire process. The user cannot turn low-quality metadata into top-notch documentation; nevertheless, its FAIRness is improved by recording any data modification during curation. Reproducibility of data harmonization is ensured by following step by step the track described in the GLP-compliant report of ESPERANTO.

## 4 Conclusion

ESPERANTO supports metadata harmonization and integration in a flexible and user-friendly fashion, allowing tailored analytical procedures and detailed GLP-compliant reporting regardless of the type and level of expertise of the user. Supplementary File S1 shows the case study evaluation of publicly available TGx metadata.

## Supplementary Material

btad405_Supplementary_Data

## Data Availability

The source code of ESPERANTO is publicly available at the following GitHub repository: https://github.com/fhaive/esperanto.
