# Supplementary material for: ESPERANTO: a GLP-field sEmi-SuPERvised toxicogenomics metadAta curatioN TOol
Source: Bioinformatics. 2023 Jun 24;39(6):btad405. doi: 10.1093/bioinformatics/btad405 (PMC10313344; doi:10.1093/bioinformatics/btad405)
Supplement: btad405_Supplementary_Data [file btad405_supplementary_data.zip › S2_Esperanto_Supplementary_File_S2.docx]

**Supplementary File S2**

**Integrating ESPERANTO in Nextcast framework**

Nextcast is a software suite containing different packages for comprehensive toxicogenomics data analysis (Serra *et al.* 2022).

The purpose of the following text is to showcase ESPERANTO potential integration into Nextcast pipelines.

As described in the paper, ESPERANTO harmonizes an uncurated dataframe-shaped phenodata with the support of a reference vocabulary. The output consists of the curated file, an enriched version of the vocabulary and a meticulous GLP/no GLP report of the process.

Therefore, by performing a GLP-compliant data curation, ESPERANTO works efficiently in combination with any other Nextcast tool processing dataframe-shaped phenodata.

In **Table 1**, sub-pipelines involving ESPERANTO are shown, in combination with a short description of the tasks performed by the interacting tools. The flexibility of ESPERANTO ensures the user the possibility to contrive and combine Nextcast packages into pipelines different from those enlisted here, if more suitable for other research aims.

For more details about the Nextcast tools, please consult Serra *et al.* 2022.

**Table 1.** Potential applications of ESPERANTO in synergy with Nextcast tools. Red indicates an input, dark blue Nextcast tools, light blue the type of output of the upstream tool, yellow represents ESPERANTO.

| **Interacting tool** | **Sub-pipeline** | **Tasks of the interacting tool** | |  |
| --- | --- | --- | --- | --- |
| **eUTOPIA** | 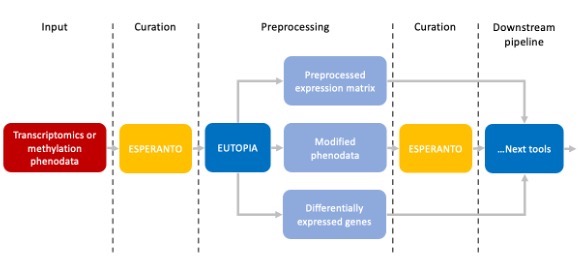 | **Preprocessing & statistical analysis of omics data**  - Raw data  quality check  - Normalization  - Batch effect  estimation &  mitigation  - Annotation  - Differential  analysis  - Data  exploration | |  |
| **BMDx** | 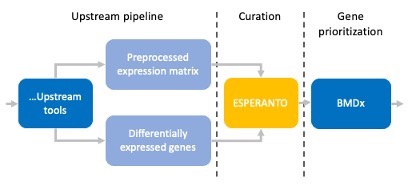 | | **Benchmark dose analysis of omics data**  - Gene filtering  - Model  selection  - Doses  estimation  - Functional  annotation | |
| **TinderMIX** | 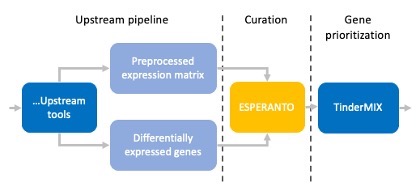 | **Assessment of dose-dependet molecular alteration at multiple times**    - Gene filtering  - Model  selection  - Prediction of  gene  deregulation - Activated  genes  identification  - Points of  Departure  estimation | |  |

In order to provide a full overview, **Figure 1** shows the example of a complete pipeline set for biomarker discovery from toxicogenomics data (adapted from Serra *et al.* 2022).


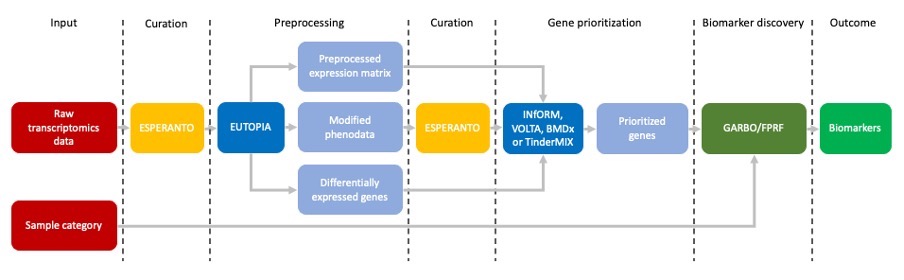


**Figure 1.** Example of complete Nextcast pipeline for biomarker identification. ESPERANTO curates the data preprocessed by EUTOPIA. Since the latter adds new columns to the phenodata, ESPERANTO is called also downstream to eUTOPIA to process the output files provided as input to BMDx or TinderMIX, which will identify a set of biomarkers. Either the complete roster of genes or just the retrieved set can be supplied to the feature selection algorithm (GARBO or FPRF) responsible to ascertain the minimal set of predictive biomarkers.

**Reference**

Serra A, Saarimäki LA, Pavel A *et al.* Nextcast: A software suite to analyse and model toxicogenomics data. *Computational and Structural Biotechnology Journal* 2022;**20**:1413–26.
