# Supplementary material for: ESPERANTO: a GLP-field sEmi-SuPERvised toxicogenomics metadAta curatioN TOol
Source: Bioinformatics. 2023 Jun 24;39(6):btad405. doi: 10.1093/bioinformatics/btad405 (PMC10313344; doi:10.1093/bioinformatics/btad405)
Supplement: btad405_Supplementary_Data [file btad405_supplementary_data.zip › S1_case_study_Evaluation_of_ESPERANTO.docx]

**Evaluation of ESPERANTO**

**Case study based on the curation of multiple**

**single datasets and their integration**

# Aims

ESPERANTO is a tool designed to ensure a GLP-compliant (Good Laboratory Practice) streamlined and standardized harmonisation of toxicogenomics (TGx) metadata. The process, carried in an user-friendly interface, results in curated datasets characterized by higher FAIR-ness. The tool offers comprehensive documentation that tracks all modifications and operations executed on the datasets. Additionally, in GLP mode, the user is required to add a mandatory comment to every operation.

ESPERANTO also manages the integration of different single curated datasets in a consistent fashion, creating the best premises to generate high-quality insights from the data.

# Brief random notes about ESPERANTO

This document does not provide a detailed description of how ESPERANTO functions. For this purpose, the link to the updated and detailed User Guide can be found in the readme at <https://github.com/fhaive/esperanto>.

ESPERANTO´s performance is based on the cross-comparison between the dataset(s) and a reference *ad hoc* vocabulary. Each curation round allows the user to enrich and update the vocabulary previously used.

The user is encouraged to think of both dataset and vocabulary files in terms of data tables, with “***labels***” naming the columns, and "***contents***” in the different cells.

The vocabulary is build as a nested synonym vocabulary, where a key reference label is linked to label synonyms and to a series of key reference contents. In turn, each of the latter is associated with its synonyms.

# Evaluation strategy

To test ESPERANTO’s efficiency, we evaluated its performances in two different scenarios. At the start, ESPERANTO generates a unique ***SHA-256* *ID*** for each running session: each file saved, and report generated result associated to that unique identifier.

Input GSE datasets, vocabulary used, saved session files and reports are provided in the *case_study_files* folder uploaded at <https://github.com/fhaive/esperanto>.

## 3.1 Single dataset

For this case study, we utilise two distinct datasets publicly available on Gene Expression Omnibus (GEO) repository (GSE199152 and GSE53845) and are relevant for Idiopathic Pulmonary Fibrosis (IPF).

The reference vocabulary to upload can be the starting version provided with this tool, or a pre-existing customized dictionary from the user if they have the same structure.

The starting reference vocabulary can be considered empty since the implemented filler row is removed as soon as the vocabulary starts to be used and populated.

At the same time, the curation of each dataset provided a list of label and content potential candidates: the user was asked to evaluate whether incorporate them into an updated version of the uploaded vocabulary.

The cross comparison of the following dataset will be performed with the updated dictionary.

GLP mode was not activated to privilege the operational linearity for the descriptive purpose of this document, but any performed operation on the data was recorded in the procedural track report. ESPERANTO generates a unique sha-256 ID that identifies all the files and reports of the same session.

Despite the GLP/procedural track report, ESPERANTO documents different aspects of the curation through several reports that guide any future user to replicate the same curated outcome obtained by the original curator.

Regarding the vocabulary update, three different reports identify the candidates accepted, those discarded and those that currently present issues to revise in a second session.

After each curation round and the correspondent potential incorporation of new-entry candidates, we evaluated:

1. the enrichment of the reference vocabulary in terms of new entries incorporated
2. the GLP-fication of the curation by providing a detailed report regarding the operative pipeline.

## 3.2 Integration of multiple datasets

In the second scenario, we considered the integration of the two datasets previously curated, using the last updated version of the vocabulary to categorise the entries of the integrated table and speed up the harmonisation quality check. As for single datasets, GLP/procedural track is one of the reports that composes the documentation of the analysis. In particular, documentation will be generated to record “consistent” as well as problematic entries to revise in a second curation session.

As for single datasets, we evaluated:

1. the merging of the curated dataset into a main harmonised table
2. the GLP-fication of the integration process by supplying a detailed report documenting the operations performed.

# Curation of Single Datasets

## The following tables belong to ESPERANTO analysis report, where the main results of the curation are summarised. The tables presented in the following paragraphs cover the pre-curation vs post-curation condition of the dataset, the vocabulary before and after the update, and an overview of the updating process.

## 4.1 Dataset I: GSE199152

As previously mentioned, this first dataset was curated using an empty reference vocabulary.

(Sha256 ID: *e535c4690a151551d5c740ea55c0100d5ab0736a4432bf8560397443e14ca31e*)

### 4.1.1 Phenodata (I)

The current dataset required less than 30 operations to be curated (**Table 2**). Due to the empty nature of the input vocabulary (**Table 3**), the majority of operations falls under the “Duplicate Removal” and “Content Homogenization” section of ESPERANTO, where the automation is limited to implementing the operations column by column.

**Table 1.** Characteristics of pristine GSE199152.


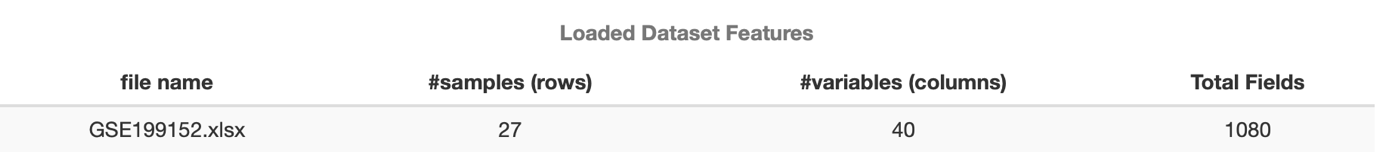


**Table 2.** Number and type of the operations required to complete the curation of the current dataset.


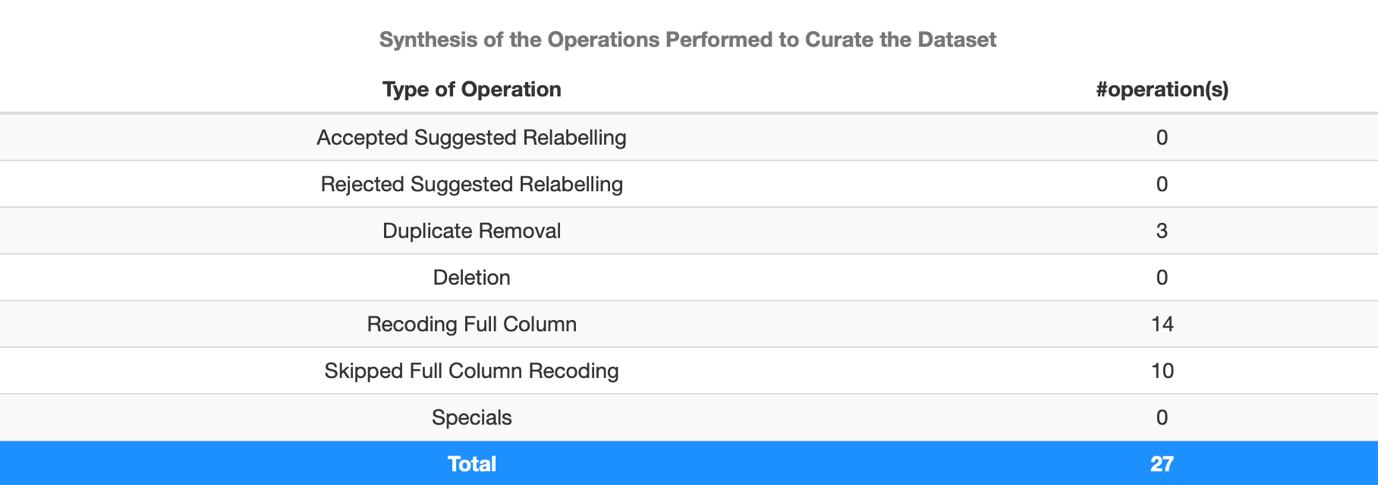


**Table 3.** Characteristics of curated GSE199152.


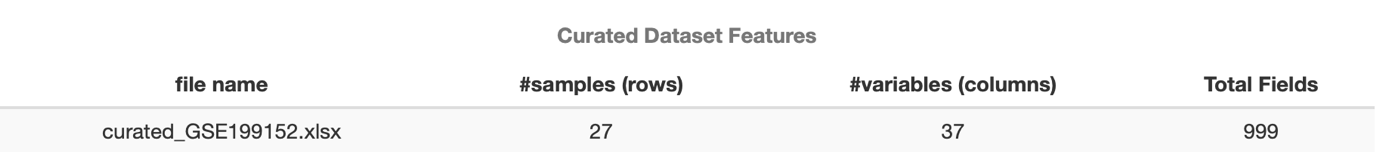


### 4.1.2 Vocabulary (I)

The curation round of GSE199152, although a relatively small dataset (**Table 1**), generated 56 entries that were positively evaluated for vocabulary enrichment (**Table 5**).

**Table 4.** Characteristics of the starting vocabulary.


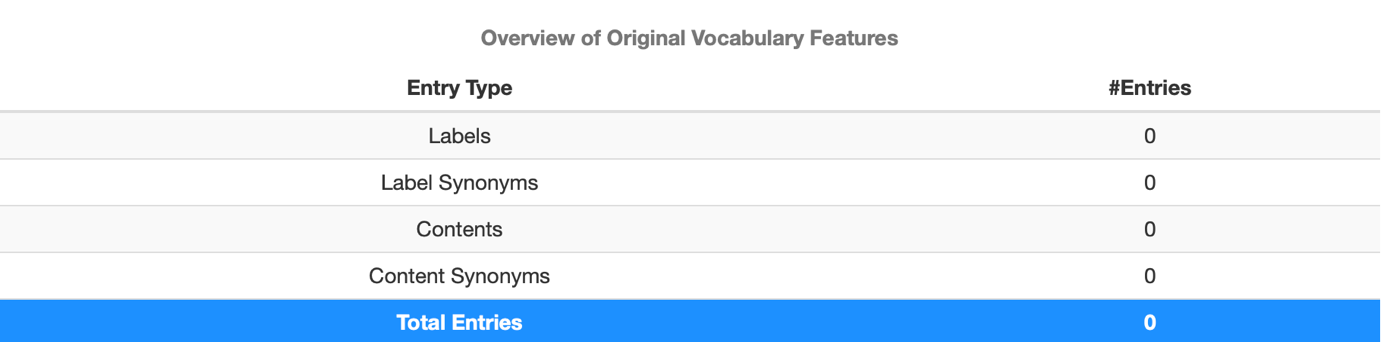


**Table 5.** Characteristics of the updated vocabulary.


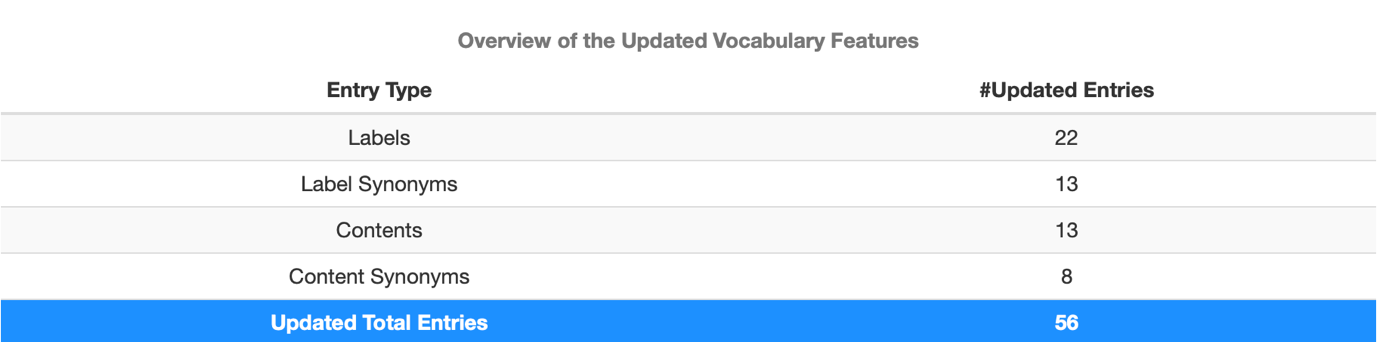


### 4.1.3 New entries vocabulary evaluation (I)

As outlined in the manual, the user can classify the candidate for vocabulary enrichment as either “Issue”, “Discard”, or “Accepted”. The outcome of this process is shown in **Table 6**. To ensure transparency and accountability, the Curator and Arbiter responsible for the classification are also recorded (**Table 7**). These measures aim to enhance the overall quality and reliability of the dataset by providing clear documentation of the curation process.

**Table 6.** Type of operations to evaluate entry candidates.


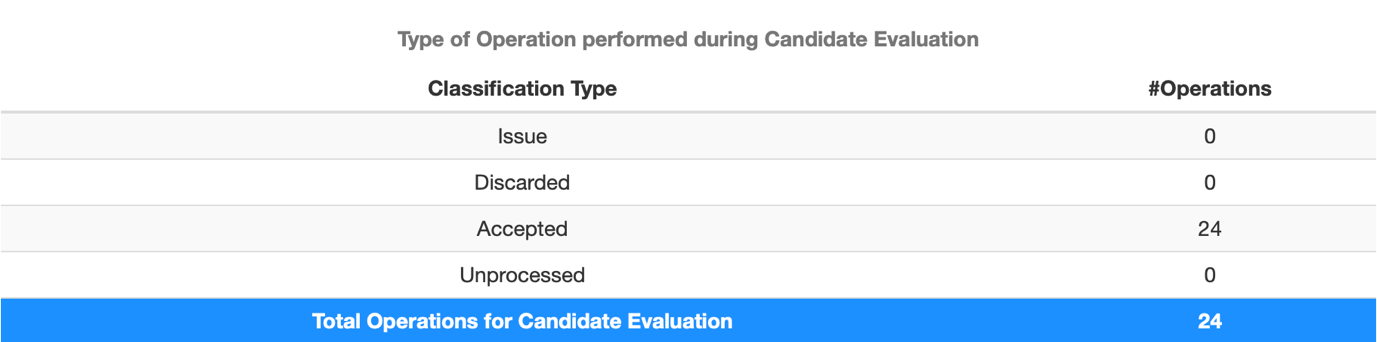


It shows only the first 6 accepted entries, but the complete reports are available in *case_study_files* folder (par. 3).

**Table 7.** First 6 entries of those accepted for vocabulary enrichment.


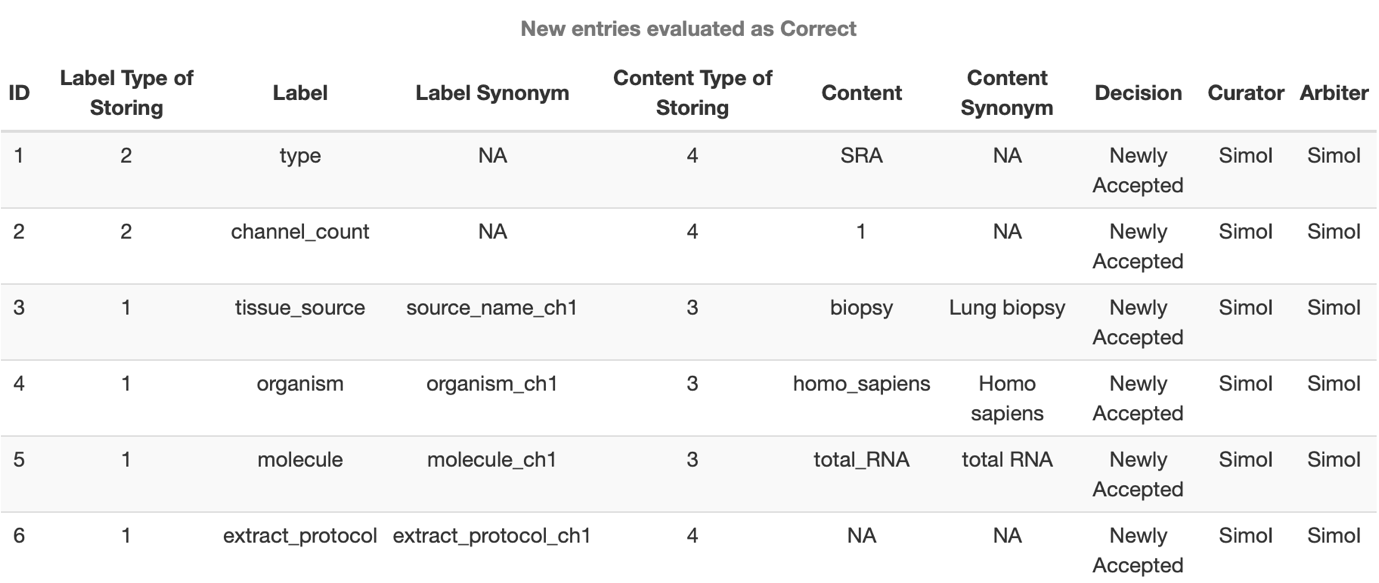


### 4.1.4 GLP-fication of the curated dataset (I)

**Table 8** shows part of the complete procedural track report available in *case_study_files f*older (par. 3). It lists the operation performed and the classification of the recoded entries as potential candidate for vocabulary enrichment.

The curation session has improved the FAIR-ness of the dataset. In particular, the procedural track is the way ESPERANTO ensures the reuse of the dataset and the reproducibility of the harmonisation process.

The other reports highlight specific aspects of the curation process, and they also support the user in integrating the information delivered by the procedural track.

**Table 8.** Subsection of the procedural track for GSE199152 curation.


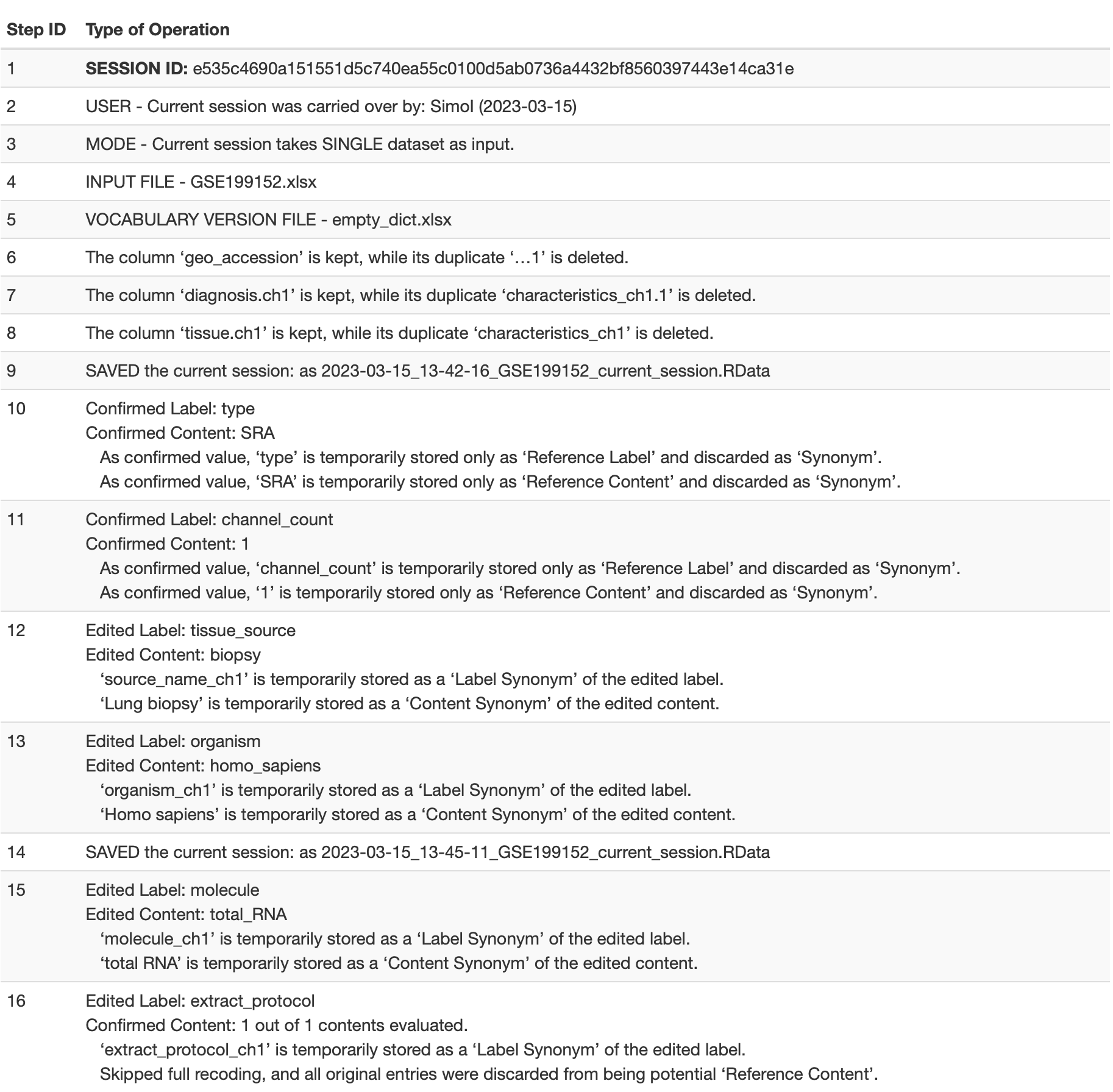


## 4.2 Dataset II: GSE53845

Compared to the previous curation round, GSE53845 will use the updated vocabulary version generated during GSE199152 harmonisation session.

(Sha256 ID: *e0d04f2dd4522fb33ad3d9f7f0411f5598d278da32f543054bd35b5900060f12*)

### 4.2.1 Phenodata (II)

Curation of GSE53845 required 54 steps to appear as a harmonised version lighter of the 20% of the original entries (**Table 9** and **Table 11**).

**Table 9.** Characteristics of pristine GSE53845.


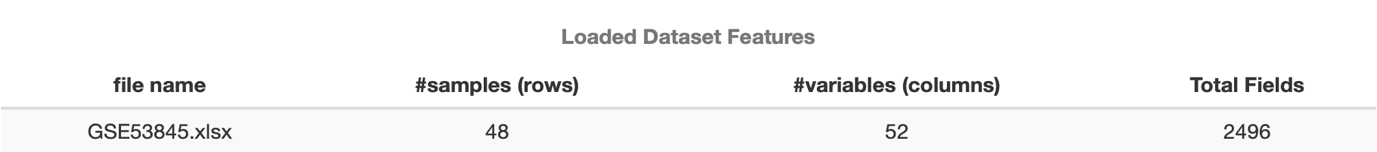


The type of operations needed to curate GSE53845 is influenced by the increased complexity of the vocabulary. Compared to par. 4.1, the user now authorized the relabelling suggestions automatically proposed by ESPERANTO (**Table 10**).

**Table 10.** Number and type of the operations required to complete the curation of the current dataset.


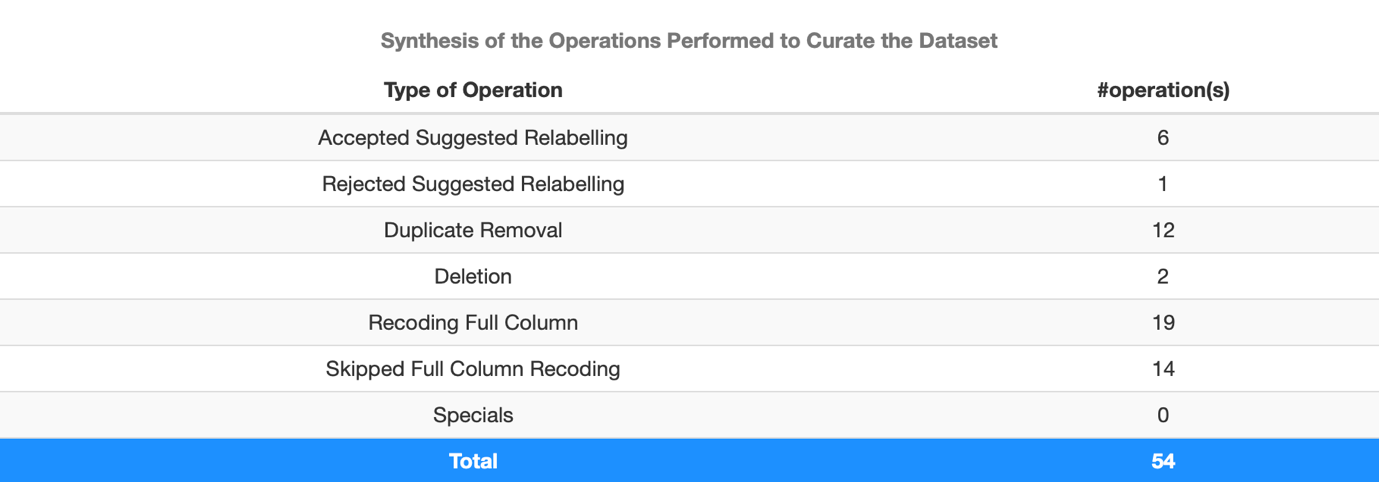


In fact, in the long run, the vocabulary enrichment will constantly reduce the active modification of the entries by the user, privileging the automatized retrieval of the reference label/content from the reference vocabulary and limiting the user intervention to supervision.

**Table 11.** Characteristics of curated GSE53845.


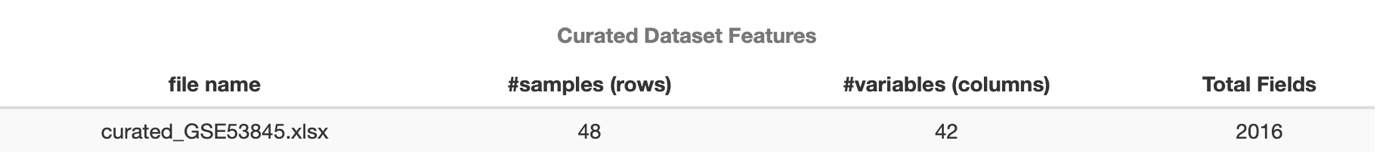


### 4.2.2 Vocabulary (II)

By using as reference vocabulary the one originated by the curation of GSE199152 (**Table 12**), harmonisation of GSE53845 was able to generate other 50 accepted new entries (**Table 13**). 34% of them are new labels establishing new categories, 24% new contents and 42% label/content synonyms.

**Table 12**. Characteristics of the input vocabulary.


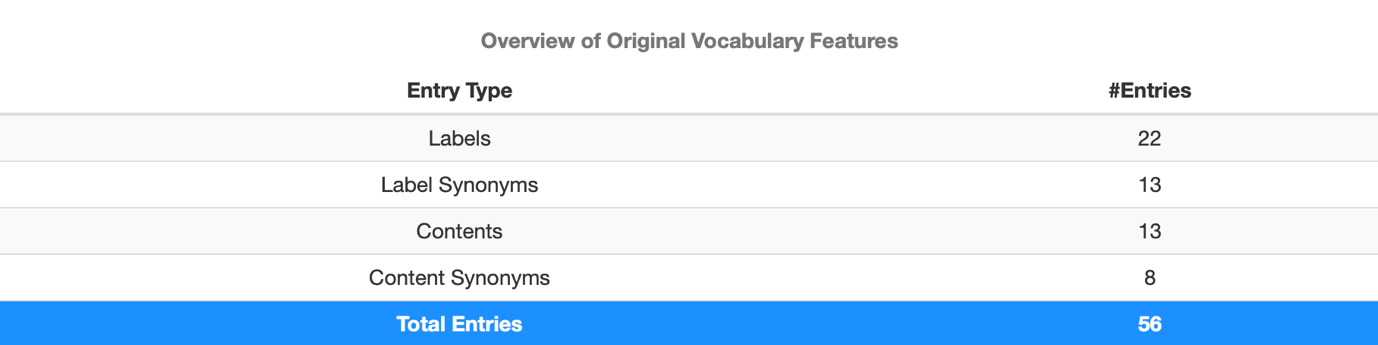


The motivation behind curation is reflected in the increase in the number of entries. Although data in GEO are presented in a certain format, there is always some degree of “subjectivity”, which leads to the identification of new candidates for vocabulary after each round of curation.

**Table 13.** Characteristics of the updated vocabulary.


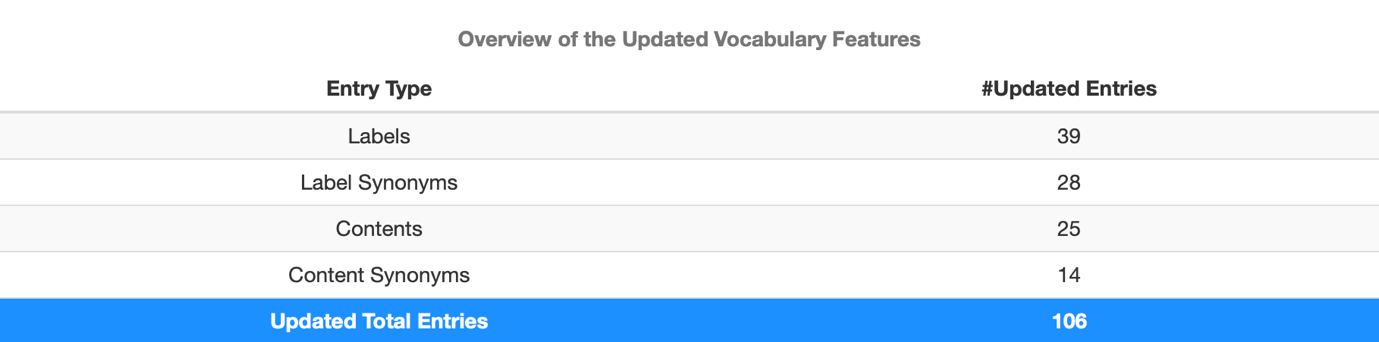


### 4.2.3 New entries vocabulary evaluation (II)

As previously mentioned in par. 4.1.3 and outlined in more detail in the manual, the user can classify candidates for vocabulary enrichment as “Issue”, “Discard”, or “Accepted”. The result of that process is shown in **Table 14**. Curator and Arbiter are also recorded allowing users to trace the origin and classification of each entry (**Table 15**).

**Table 14.** Type of operations to evaluate entry candidates.


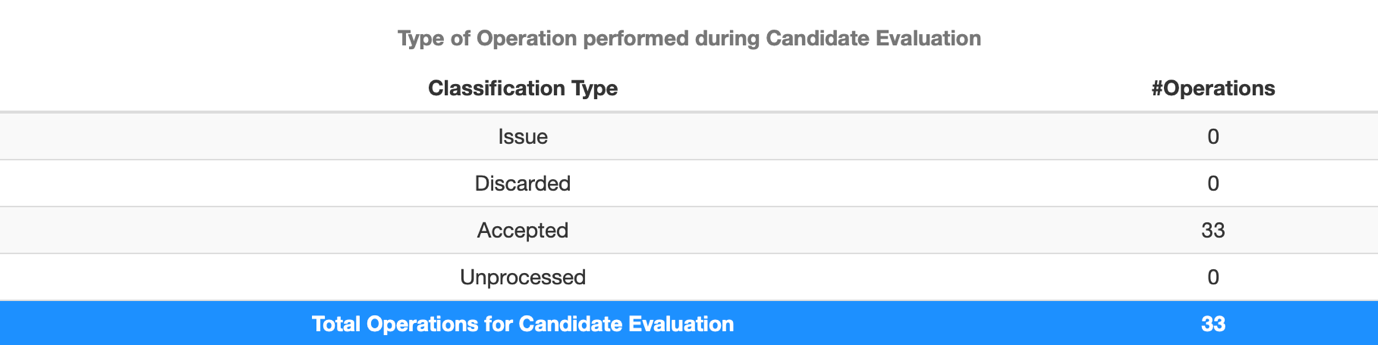


The complete documentation is available in the *case_study_files* folder (par. 3).

**Table 15.** First 6 entries of those accepted for vocabulary enrichment.


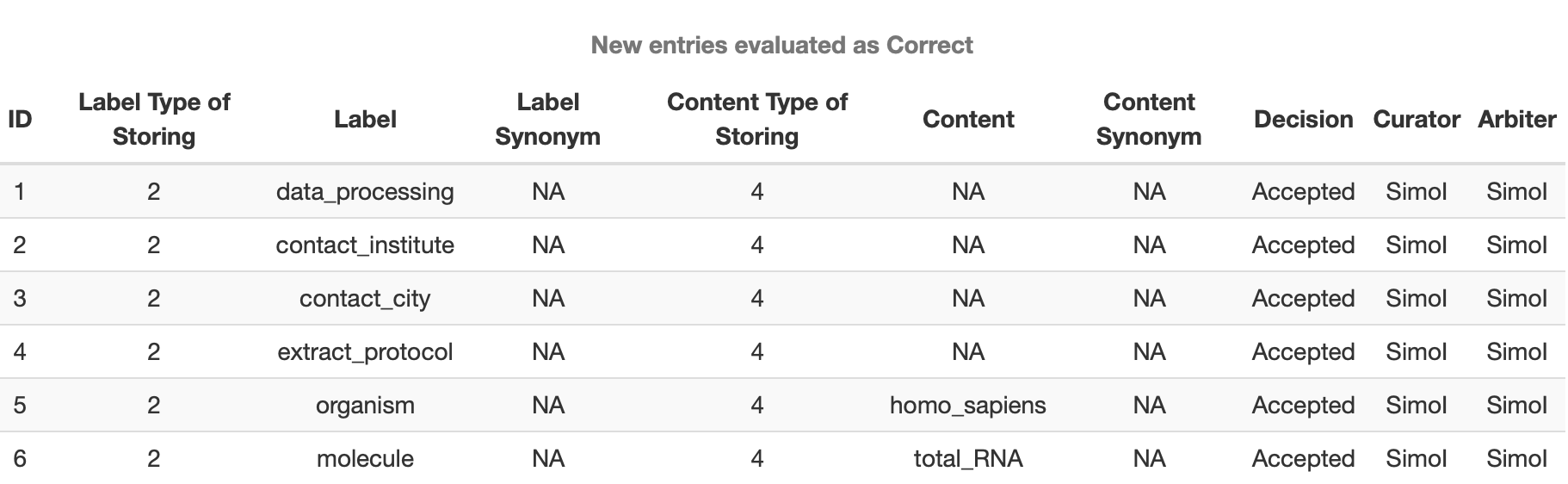


### 4.2.4 GLP-fication of the curated dataset (II)

**Table 16** shows a selection of the complete procedural track report available in the *case_study_files* folder (par. 3). This report provides a list of operations performed during the curation session and classifies the recoded entries as potential candidate for vocabulary enrichment.

Through this process, the FAIR-ness of the dataset has been significantly improved. The procedural track ensures that the dataset is reusable and the harmonisation process is reproducible through the standardized pipeline of ESPERANTO.

The additional reports highlight specific aspects of the curation process and support the user in integrating the information delivered by the procedural track reports.

**Table 16.** Subsection of the procedural track for curation session.


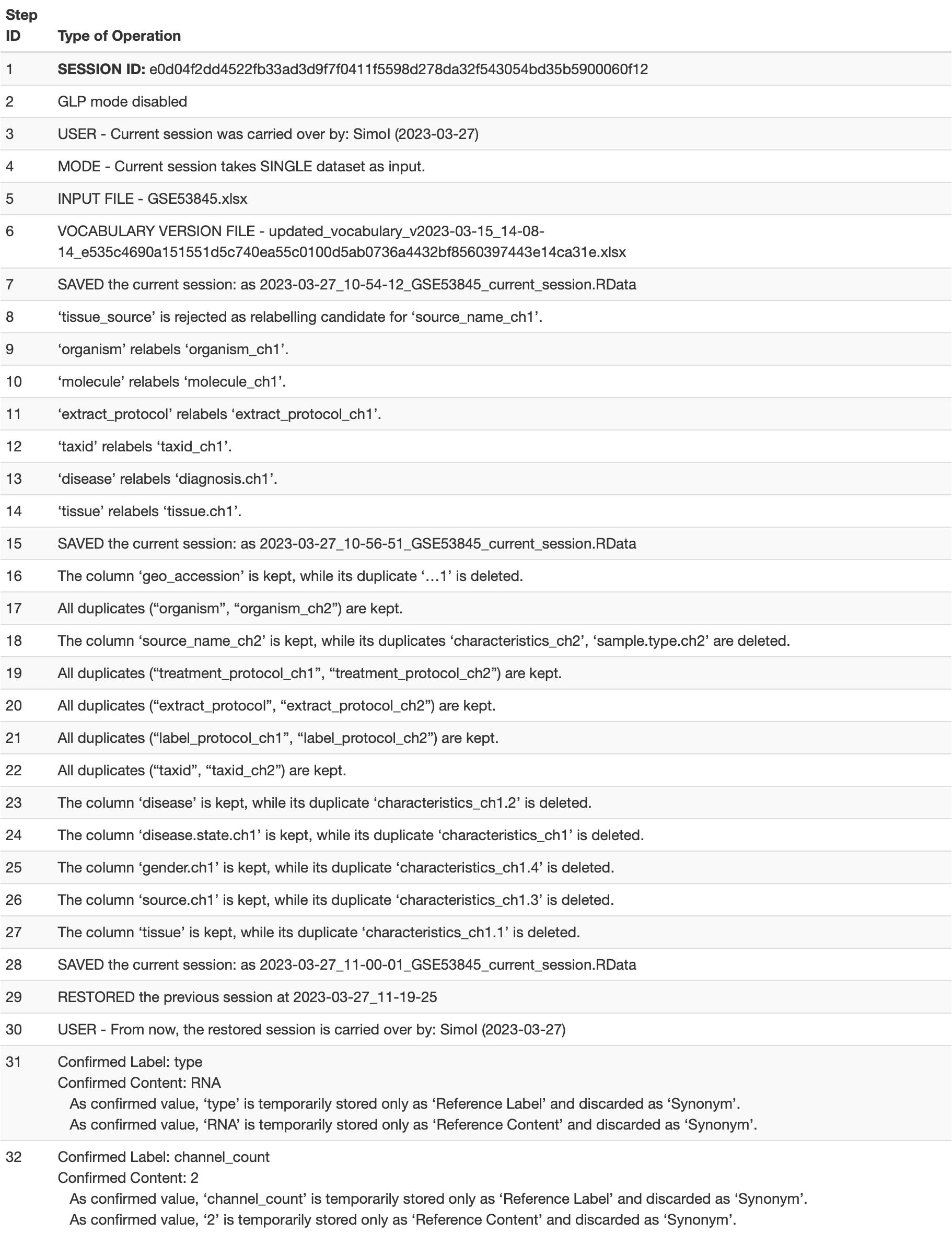


# Integration of multiple curated datasets

### 5.1 Integrated Dataset (M)

Curated GSE199152 and GSE53845 datasets were successfully uploaded and merged together into a table with 4200 fields (**Table 17**).

**Table 17.** Characteristics of single curated datasets and of integrated table.


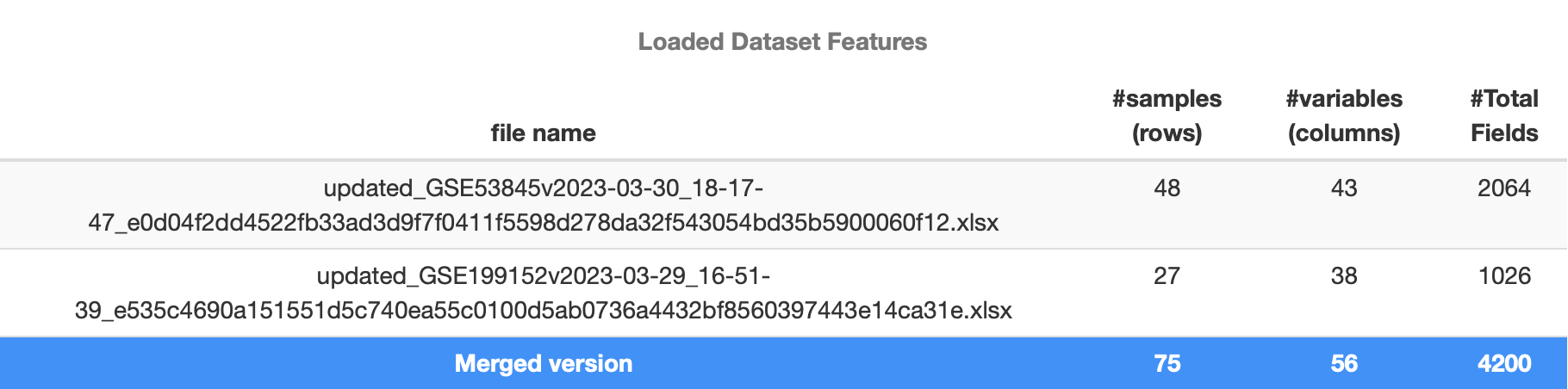


### 5.2 Vocabulary (M)

The integrated dataset was cross-compared with the latest updated version of the vocabulary (in this case the one resulting from the curation of GSE53845, shown in **Table 18**). The cross-comparison resulted in color-coded entries, with green indicating that the data labels/contents are already present in the reference vocabulary, and red to claim that they are not.

**Table 18.** Characteristics of the uploaded reference vocabulary to evaluate the integration of multiple curated datasets.


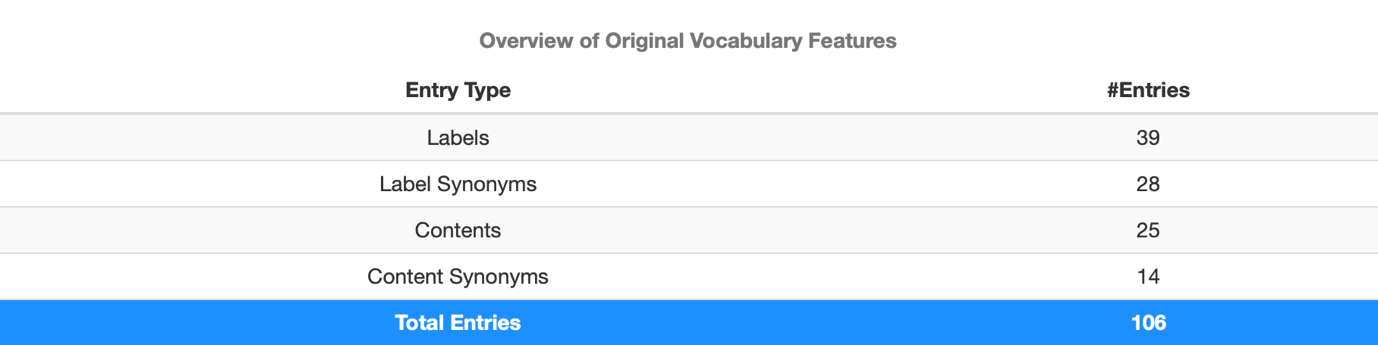


### 5.3 Integration Evaluation (M)

55 operations were needed to evaluate the quality of the integration of the datasets previously curated (**Table 19**).

**Table 19.** Type of operations to evaluate the integration of multiple curated datasets.


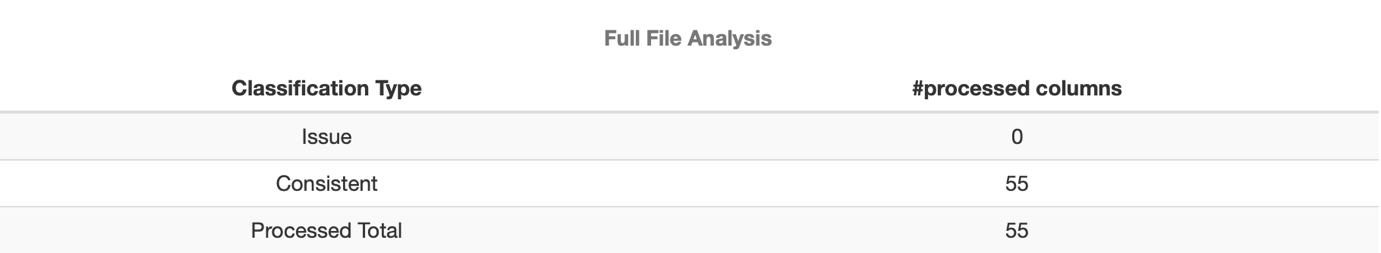


The color-coding is the base on which the user can evaluate the entries to identify coherent terms as well as those that may require additional curation at the level of individual datasets. **Table 20** presents the first 6 columns evaluated as successfully integrated.

**Table 20.** First 6 entries classified as consistent during the evaluation of the integration of multiple curated datasets.


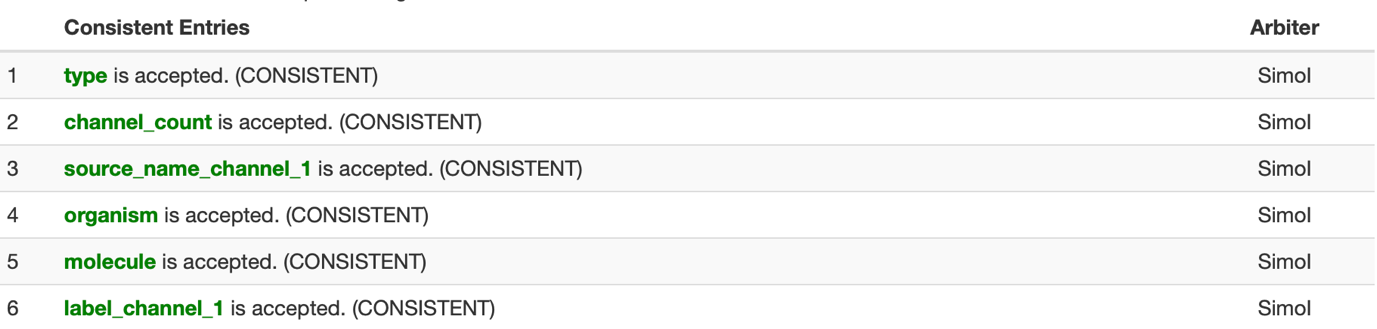


### 5.4 GLP-fication of the integration of curated dataset (M)

**Table 21** shows an extract of the complete procedural track report available in *case_study_files* folder (par. 3)

**Table 21.** Extract of the procedural track for the integration of multiple curated datasets.


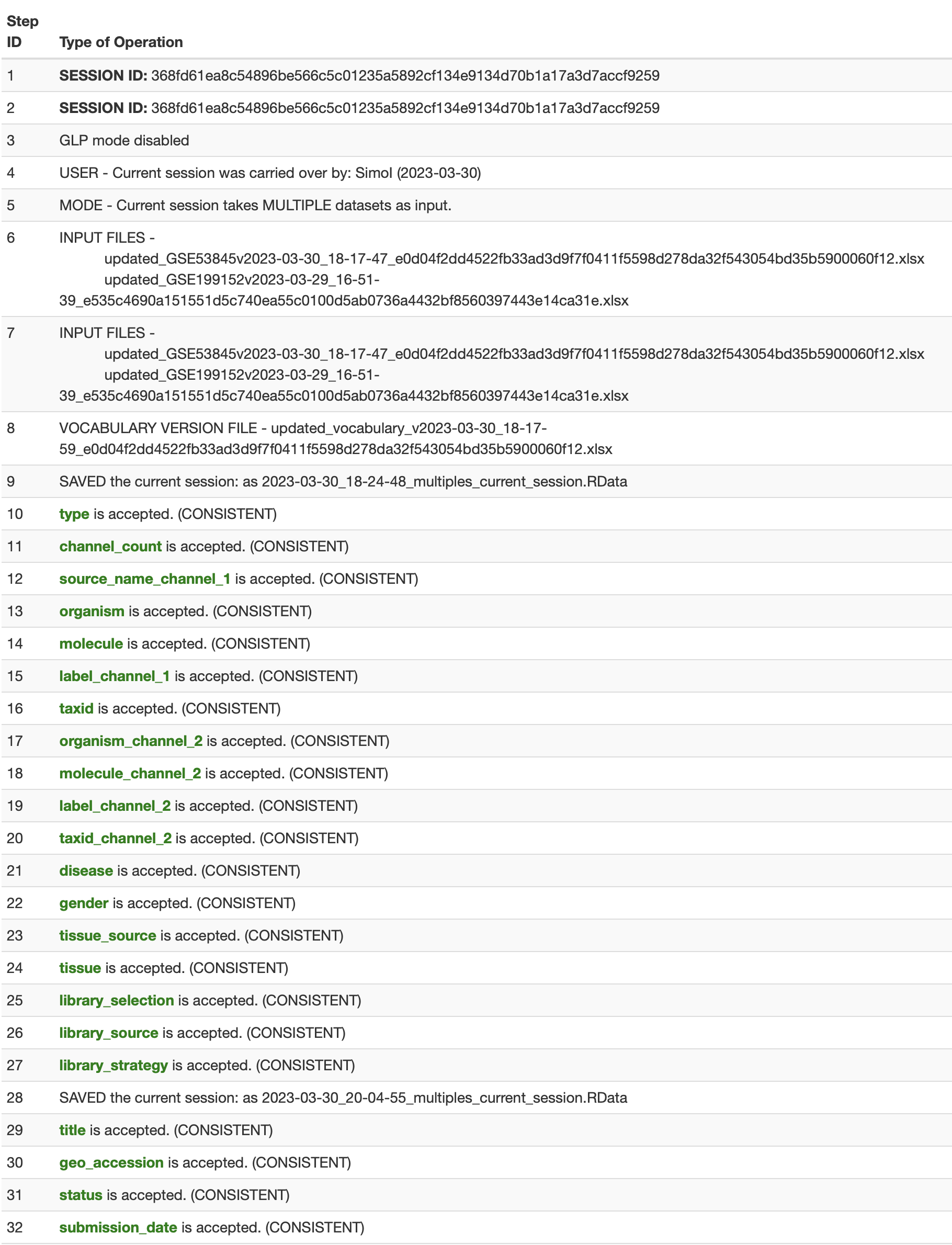


# Conclusions

The two datasets have been meticulously curated to ensure a smooth integration, an essential step towards performing further analysis.

The curation process and the entire decision-making pipeline have been well-documented to ensure reproducibility for new users.

The generated documentation provides a step-by-step guide to replicate the exact outcome achieved by the first researcher.

The GLP mode will supplement the main by providing additional information about the list of operations performed and the reasoning behind each decision made by the researcher, enabling future users to gain a deeper understanding of the applied methodology. Additionally, the vocabulary has been enriched as consequence of the curation round and subjected to an additional quality check by the user to ensure consistency and accuracy.

Overall, the curated datasets and accompanying reports provide a comprehensive resource for researchers seeking to leverage this valuable resource for data harmonisation and integration. By providing this level of documentation, ESPERANTO contributes to the dataset's reliability and reproducibility, ensuring that future analyses can be conducted with confidence.
