# Supplementary figures and images for: ESPERANTO: a GLP-field sEmi-SuPERvised toxicogenomics metadAta curatioN TOol
Source: Bioinformatics. 2023 Jun 24;39(6):btad405. doi: 10.1093/bioinformatics/btad405 (PMC10313344; doi:10.1093/bioinformatics/btad405)

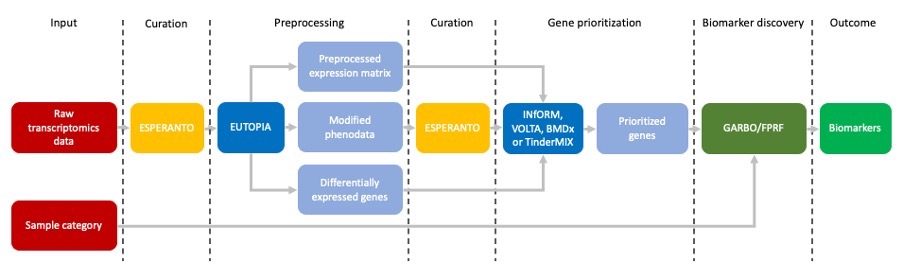

Supplement: btad405_Supplementary_Data [file btad405_supplementary_data.zip › S2_esperanto_Nextcast.jpg]

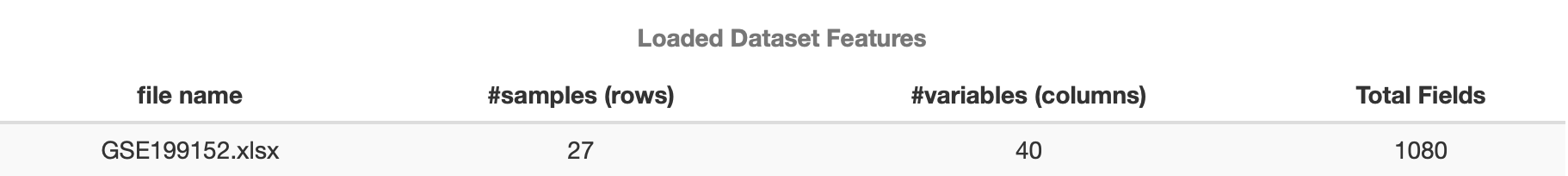

Supplement: btad405_Supplementary_Data [file btad405_supplementary_data.zip › tab01_f01_casestudy GSE199152orig dset.png]

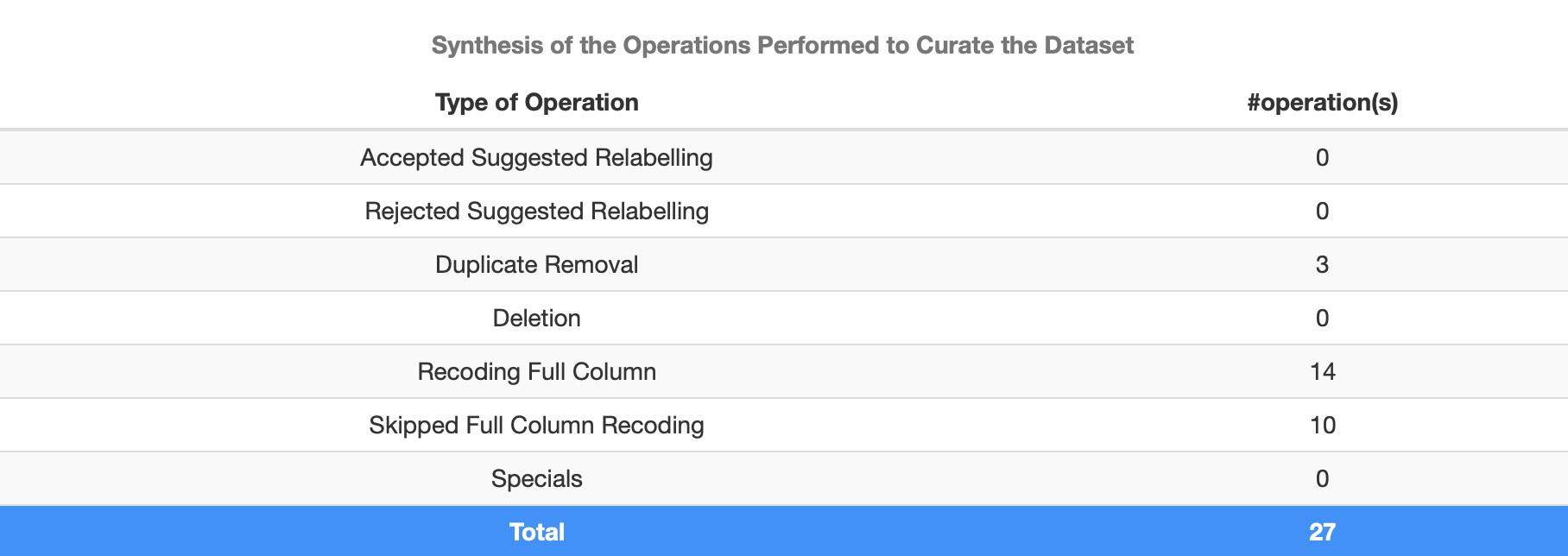

Supplement: btad405_Supplementary_Data [file btad405_supplementary_data.zip › tab02_f03_casestudy GSE199152curation ops.png]

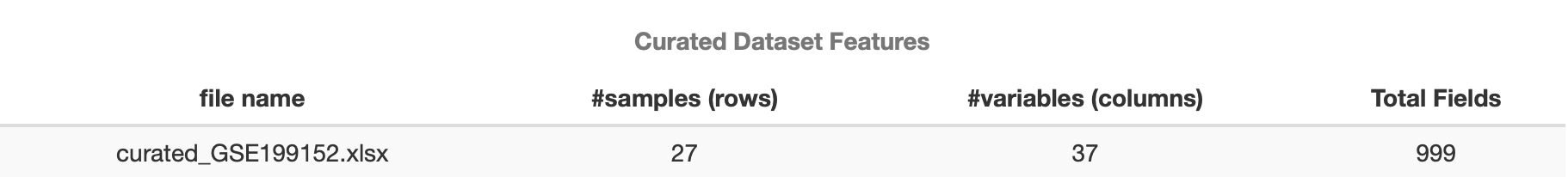

Supplement: btad405_Supplementary_Data [file btad405_supplementary_data.zip › tab03_f02_casestudy GSE199152curated dset features.png]

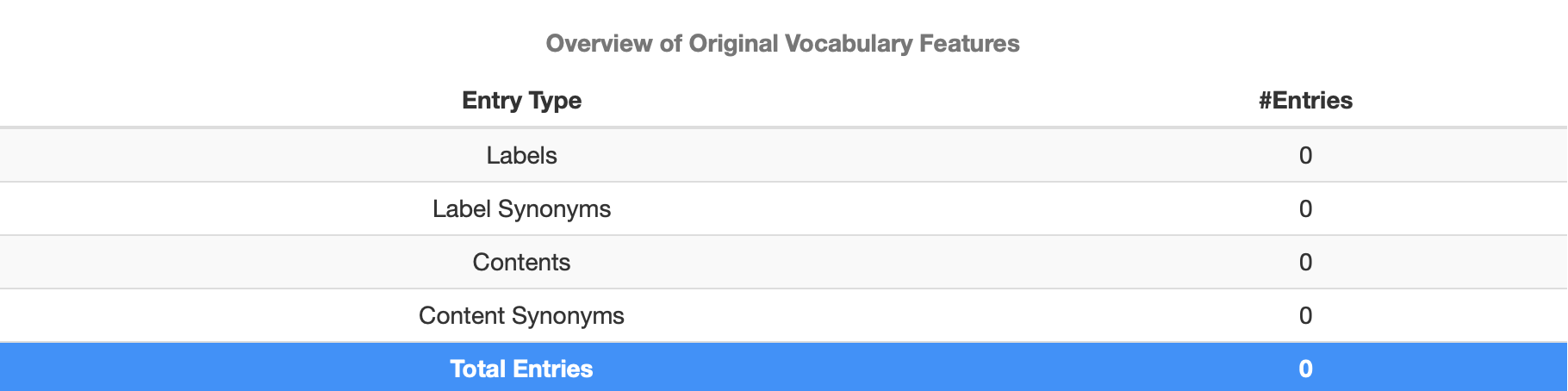

Supplement: btad405_Supplementary_Data [file btad405_supplementary_data.zip › tab04_f04_casestudy GSE199152 old voc features.png]

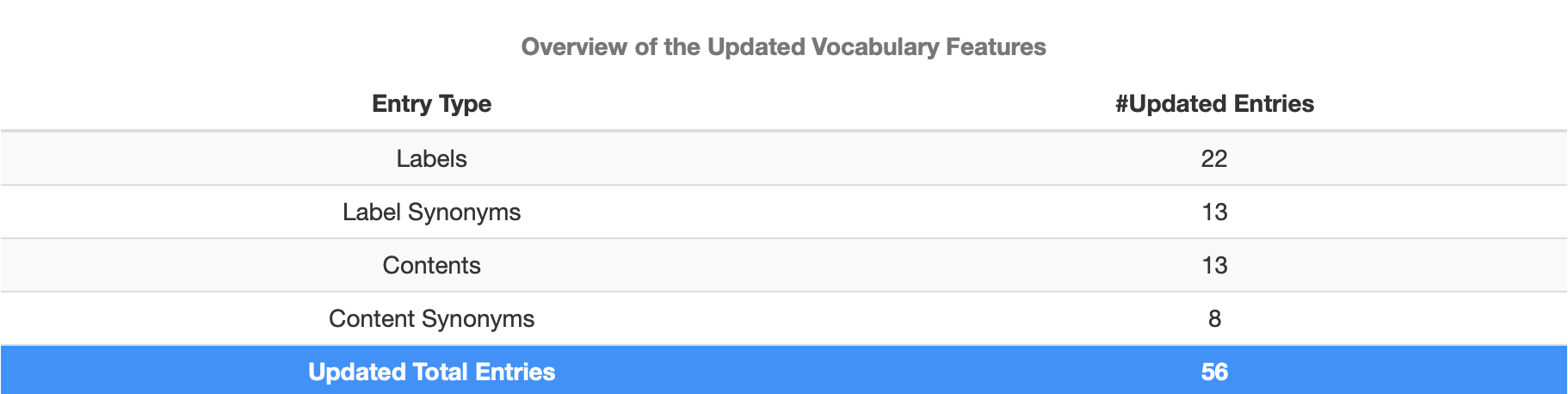

Supplement: btad405_Supplementary_Data [file btad405_supplementary_data.zip › tab05_f05_casestudy GSE199152 new voc features.png]

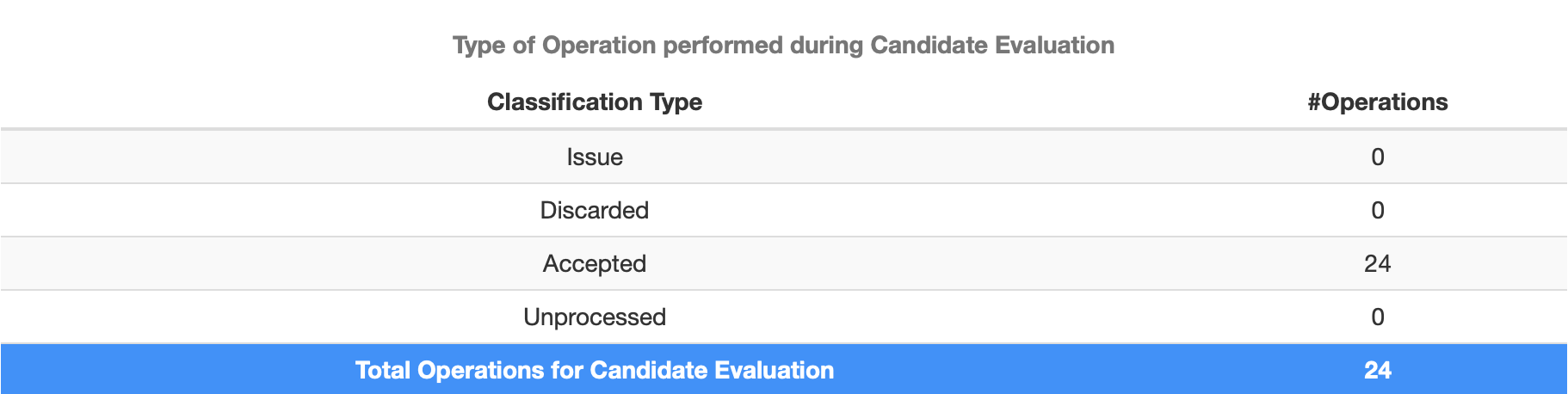

Supplement: btad405_Supplementary_Data [file btad405_supplementary_data.zip › tab06_f06_casestudy GSE199152 num ops evaluation upd voc.png]

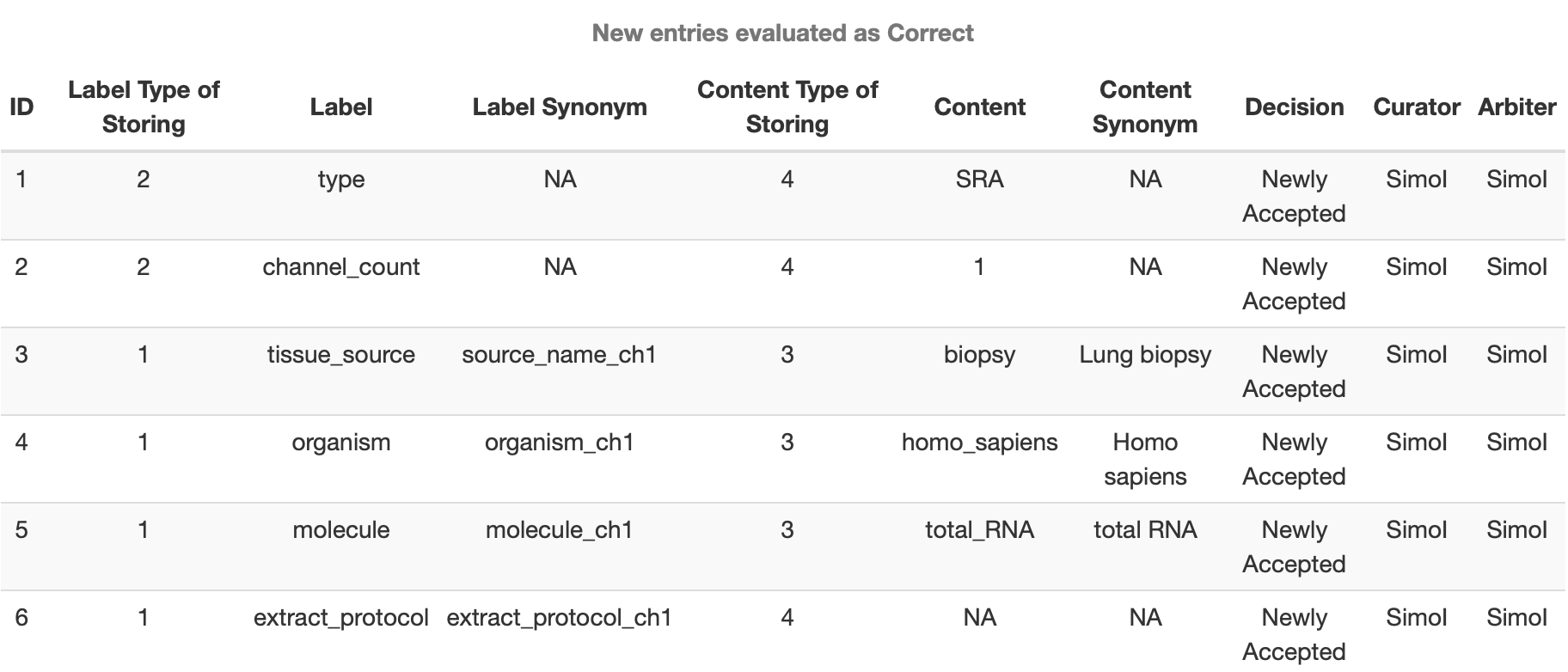

Supplement: btad405_Supplementary_Data [file btad405_supplementary_data.zip › tab07_f07_casestudy GSE199152 list accepted for voc upd .png]

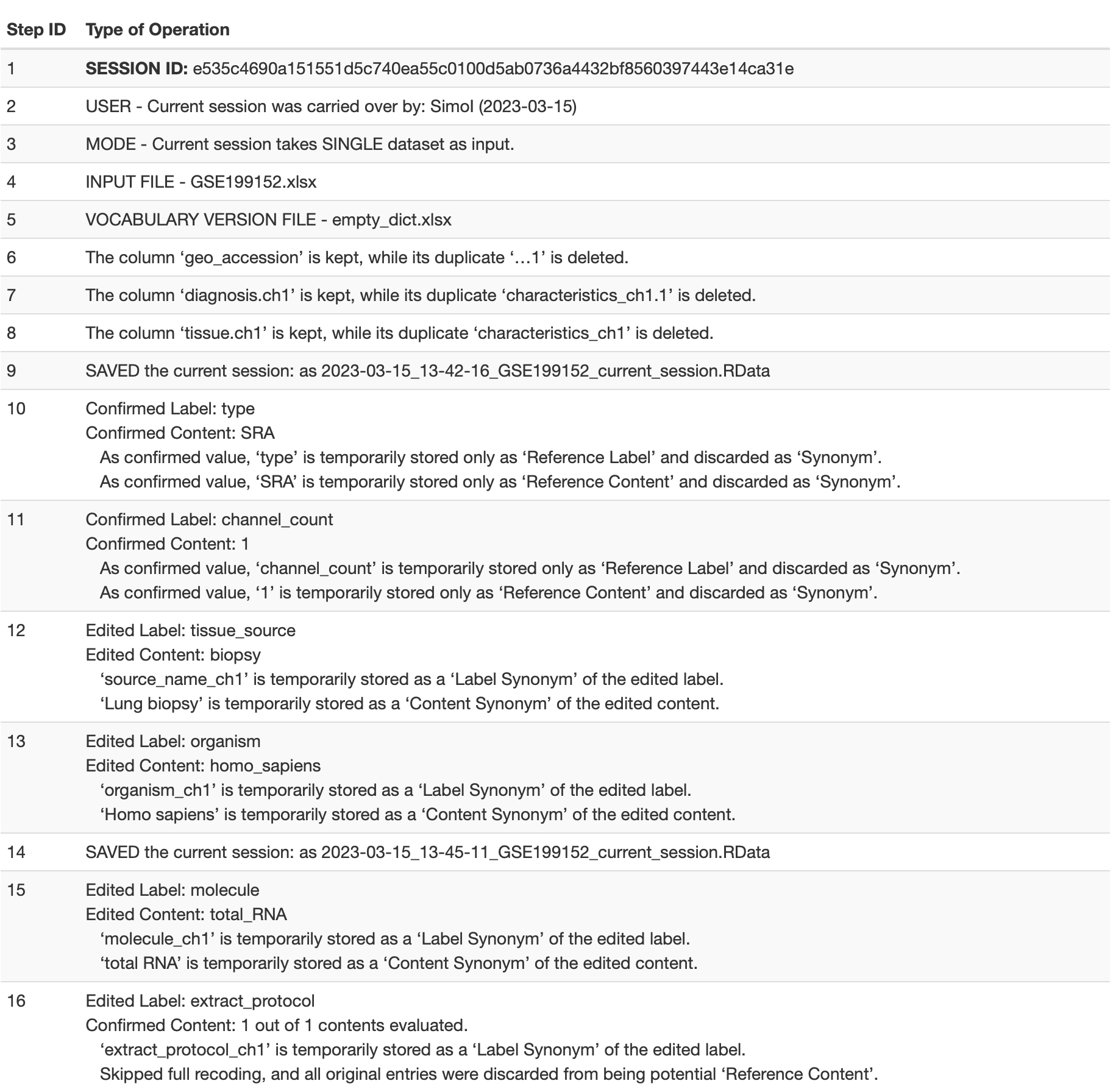

Supplement: btad405_Supplementary_Data [file btad405_supplementary_data.zip › tab08_f08_casestudy GSE199152procedure.png]

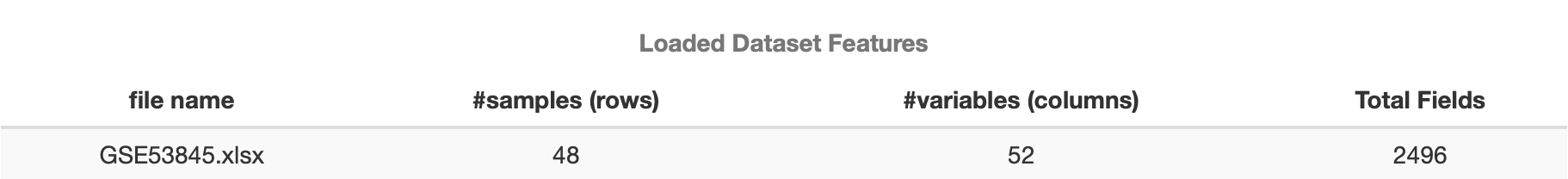

Supplement: btad405_Supplementary_Data [file btad405_supplementary_data.zip › tab09_f01_casestudy GSE53845orig dset.png]

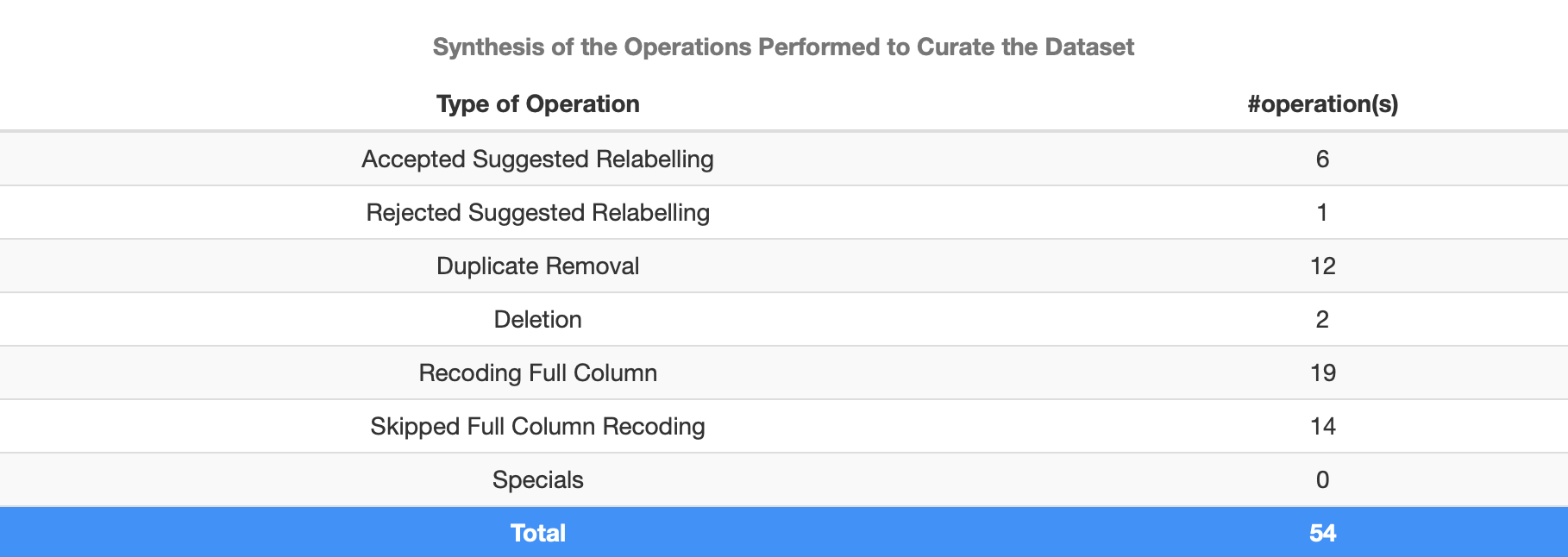

Supplement: btad405_Supplementary_Data [file btad405_supplementary_data.zip › tab10_f02_casestudy GSE53845curated dset features.png]

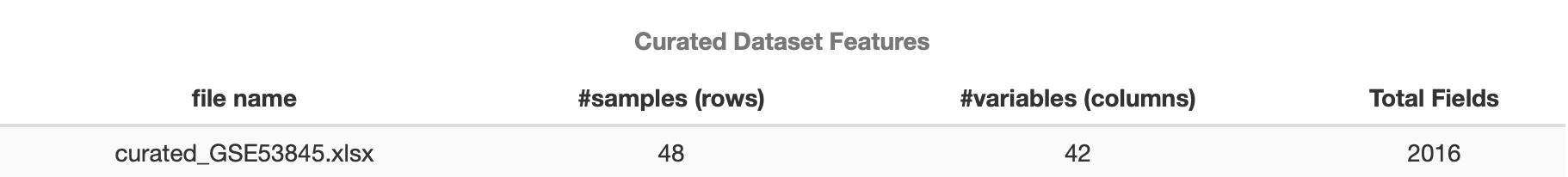

Supplement: btad405_Supplementary_Data [file btad405_supplementary_data.zip › tab11_f03_casestudy GSE53845curation ops.png]

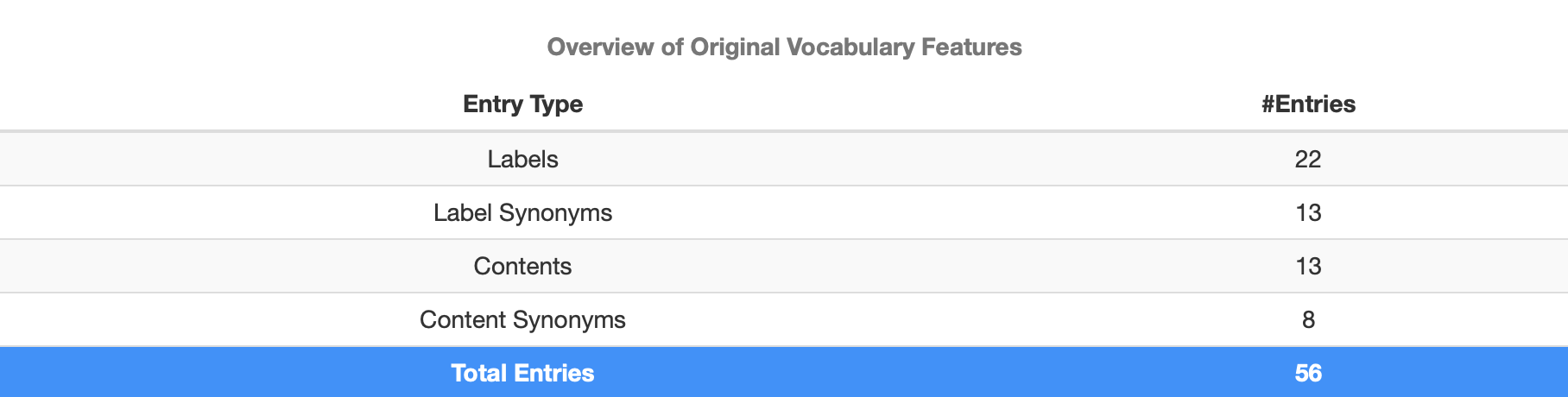

Supplement: btad405_Supplementary_Data [file btad405_supplementary_data.zip › tab12_f04_casestudy GSE53845 old voc features.png]

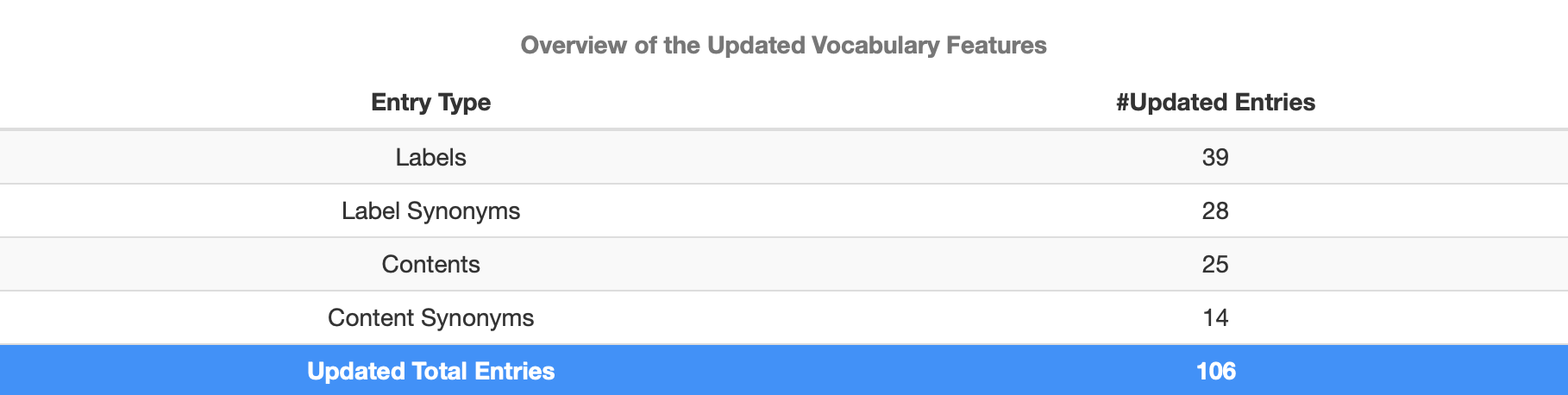

Supplement: btad405_Supplementary_Data [file btad405_supplementary_data.zip › tab13_f05_casestudy GSE53845 new voc features.png]

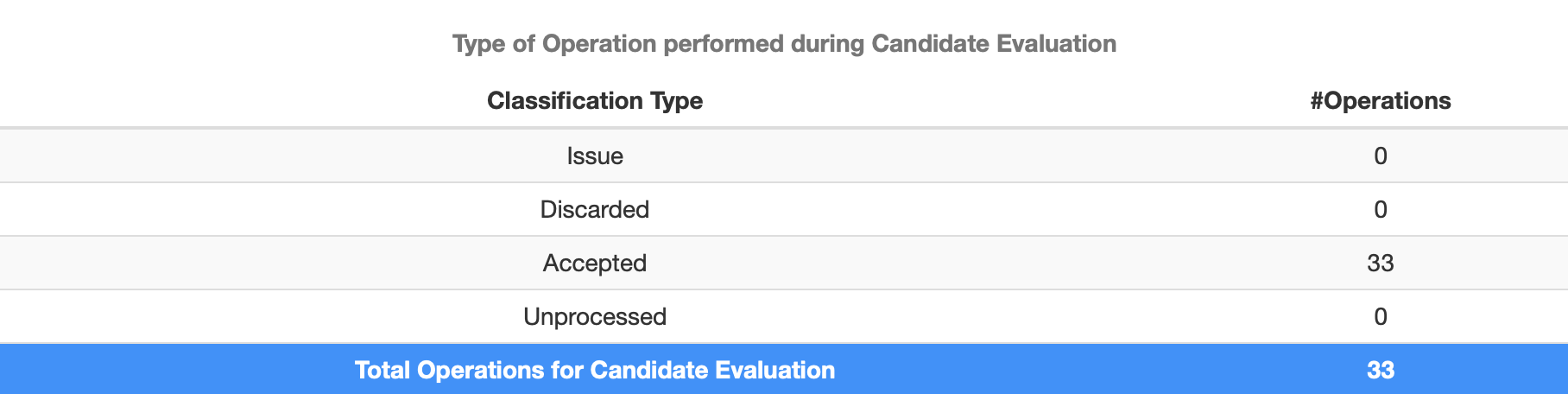

Supplement: btad405_Supplementary_Data [file btad405_supplementary_data.zip › tab14_f06_casestudy GSE53845 num ops evaluation upd voc.png]

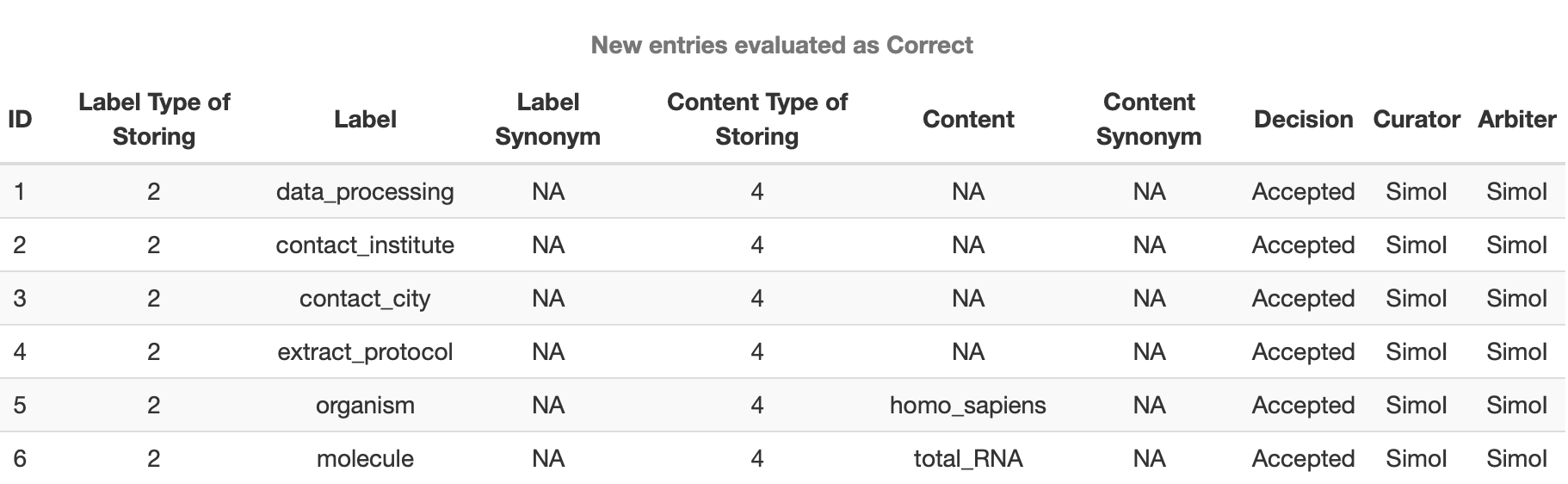

Supplement: btad405_Supplementary_Data [file btad405_supplementary_data.zip › tab15_f07_casestudy GSE53845 list accepted for voc upd .png]

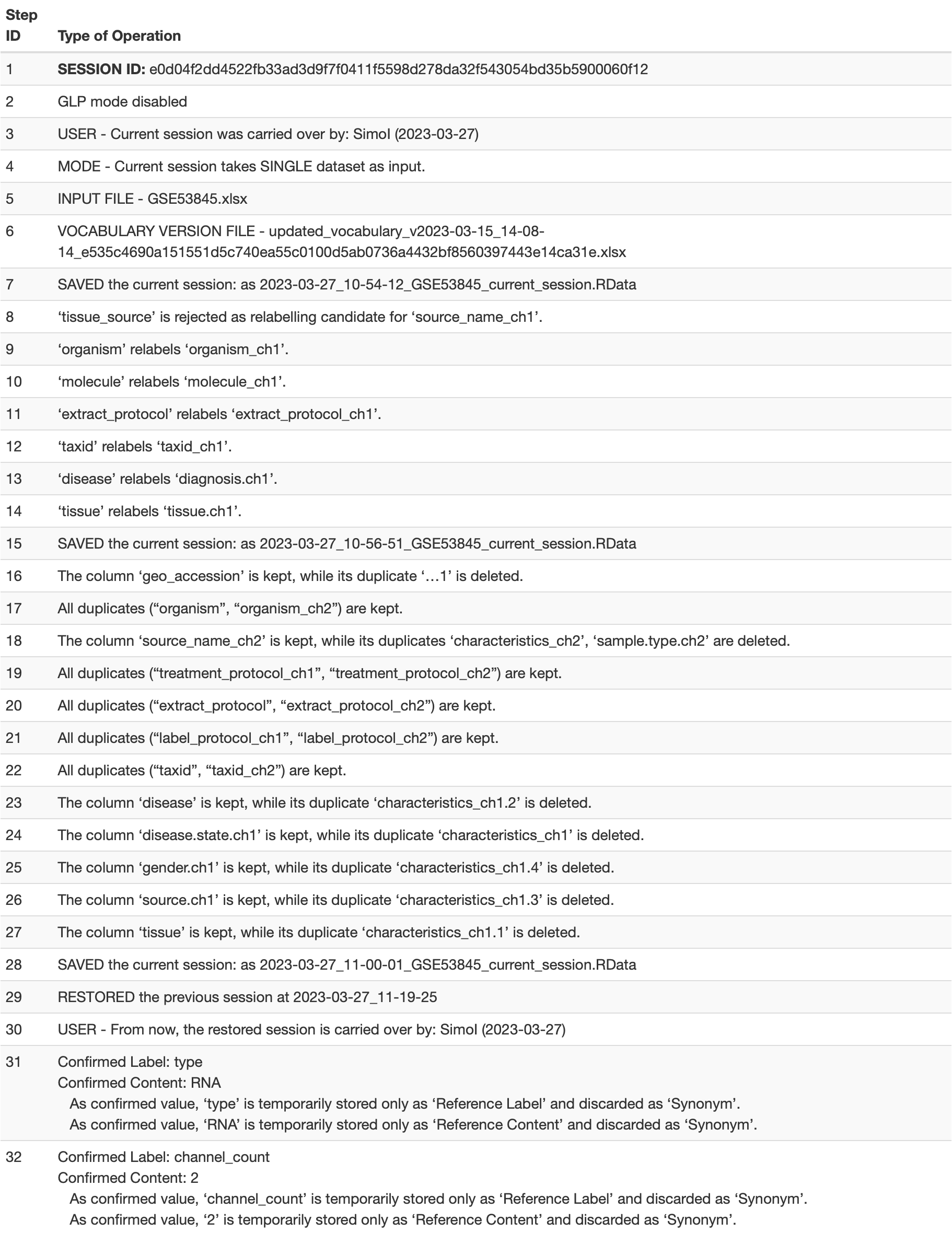

Supplement: btad405_Supplementary_Data [file btad405_supplementary_data.zip › tab16_f08_casestudy GSE53845procedure.png]

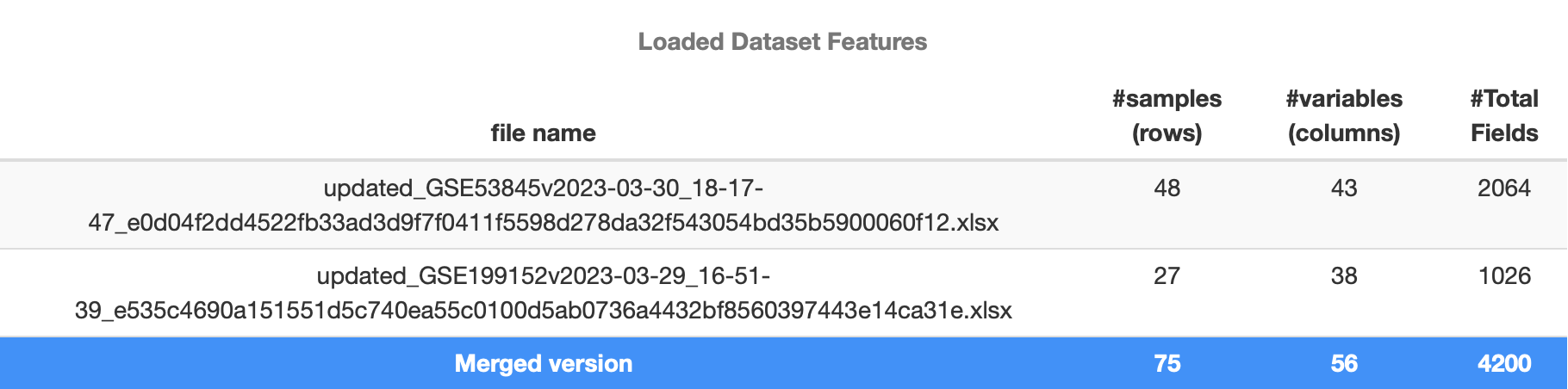

Supplement: btad405_Supplementary_Data [file btad405_supplementary_data.zip › tab17_f01_casestudy_MULTIorig dset.png]

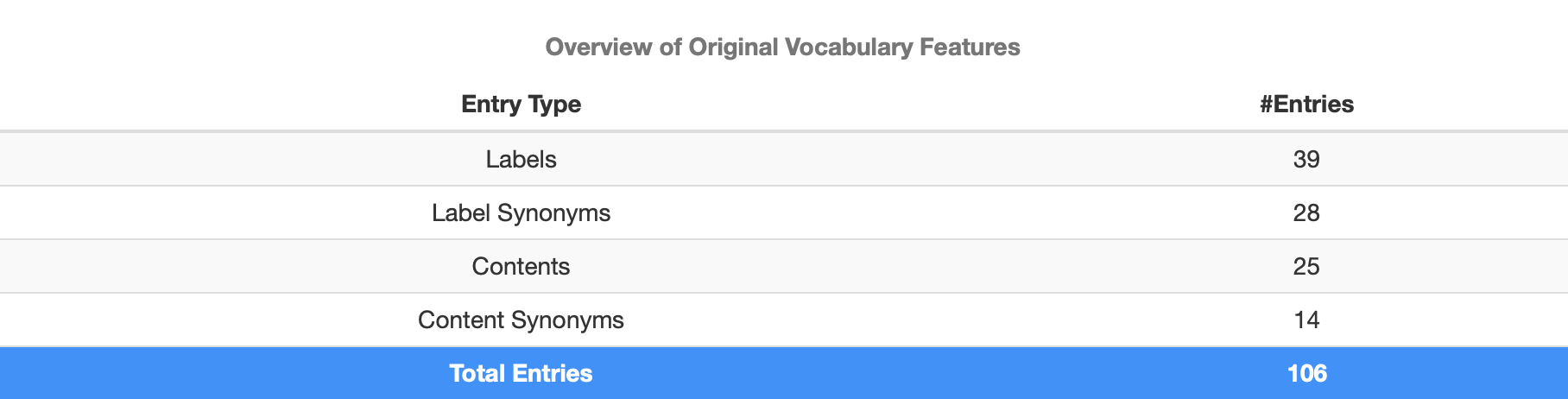

Supplement: btad405_Supplementary_Data [file btad405_supplementary_data.zip › tab18_f02_casestudy_MULTIvoc.png]

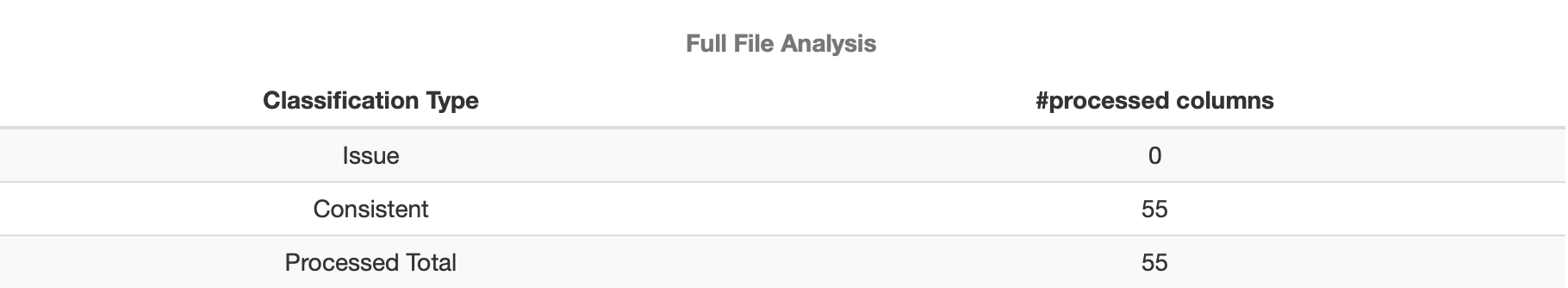

Supplement: btad405_Supplementary_Data [file btad405_supplementary_data.zip › tab19_f03_casestudy_MULTI_evaluation_integration.png]

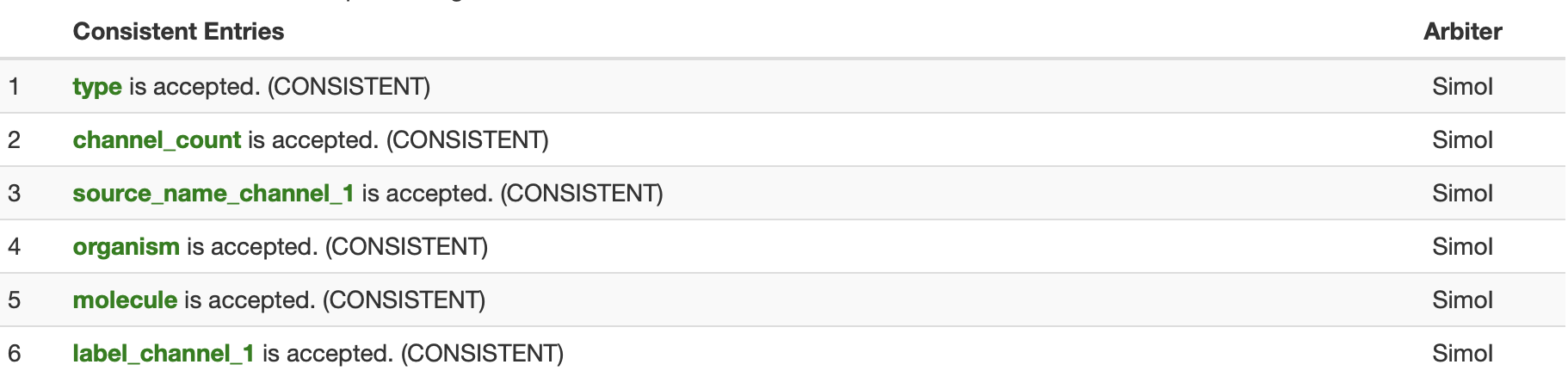

Supplement: btad405_Supplementary_Data [file btad405_supplementary_data.zip › tab20_f04_casestudy_MULTI list consistent.png]

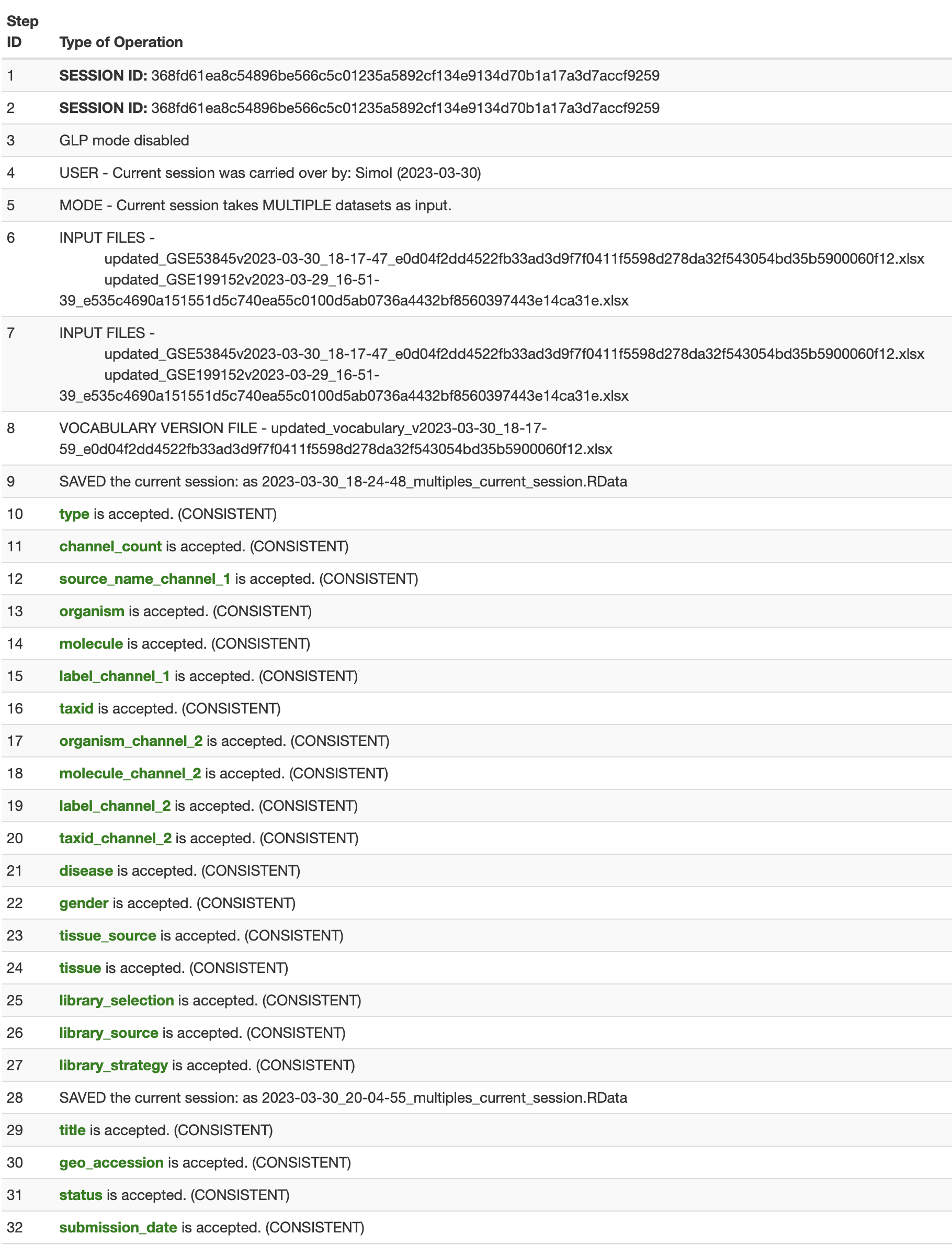

Supplement: btad405_Supplementary_Data [file btad405_supplementary_data.zip › tab21_f05_casestudy _MULTIprocedure.png]
